# Motor “laziness” constrains fixation selection in real-world tasks

**DOI:** 10.1073/pnas.2302239121

**Published:** 2024-03-12

**Authors:** Charlie S. Burlingham, Naveen Sendhilnathan, Oleg Komogortsev, T. Scott Murdison, Michael J. Proulx

**Affiliations:** ^a^Reality Labs Research, Meta Platforms Inc., Redmond, WA 98052; ^b^Department of Psychology, New York University, New York, NY 10003; ^c^Department of Computer Science, Texas State University, San Marcos, TX 78666; ^d^Reality Labs, Meta Platforms Inc., Redmond, WA 98052

**Keywords:** re-centering bias, center bias, inhibition of return, energy optimality, fixation selection

## Abstract

Humans coordinate their eye, head, and body movements to gather information from a dynamic environment while maximizing reward and minimizing biomechanical and energetic costs. However, such natural behavior is not possible in traditional experiments employing head/body restraints and artificial, static stimuli. Therefore, it is unclear to what extent mechanisms of fixation selection discovered in lab studies, such as inhibition-of-return (IOR), influence everyday behavior. To address this gap, participants performed nine real-world tasks, including driving, visually searching for an item, and building a Lego set, while wearing a mobile eye tracker (169 recordings; 26.6 h). Surprisingly, in all tasks, participants most often returned to what they just viewed and saccade latencies were shorter preceding return than forward saccades, i.e., consistent with facilitation, rather than inhibition, of return. We hypothesize that conservation of eye and head motor effort (“laziness”) contributes. Correspondingly, we observed center biases in fixation position and duration relative to the head’s orientation. A model that generates scanpaths by randomly sampling these distributions reproduced all return phenomena we observed, including distinct 3-fixation sequences for forward versus return saccades. After controlling for orbital eccentricity, one task (building a Lego set) showed evidence for IOR. This, along with small discrepancies between model and data, indicates that the brain balances minimization of motor costs with maximization of rewards (e.g., accomplished by IOR and other mechanisms) and that the optimal balance varies according to task demands. Supporting this account, the orbital range of motion used in each task traded off lawfully with fixation duration.

## Significance

Humans display remarkably precise yet flexible control of eye and body movements, allowing for a wide range of activities. However, most studies of gaze behavior use the same setup: a head-restrained participant viewing small images on a computer. Such lab studies find that people avoid looking at the same location twice, and hesitate in cases when they do. We had people perform nine everyday activities while wearing glasses with embedded eye tracking and surprisingly found that they did the opposite, often returning to what they just viewed and expediting these “return” eye movements over others. A tendency to keep the eyes centered in the head, which we speculate helps to conserve motor effort, explained these behaviors for all tasks.

Fixation selection can be described as an active inference process which maximizes reward while minimizing costs for the agent. In this framework, gaze shifts and maintenance of fixation both serve to reduce uncertainty about task-relevant properties of the environment, preventing suboptimal actions (1–4). As an example, fixating an ambiguous road sign while driving enables reading and heeding the sign’s text, “Detour,” reducing the total time taken to reach a destination (a type of reward). And because active inference takes place in a physical body, each muscular contraction and neural computation involved in generating a gaze shift, fixation, or body movement incurs specific costs. Therefore, the brain must estimate these costs and account for them when planning actions, balancing them with expected rewards and reductions in uncertainty. Indeed, simply assuming that the brain optimizes metabolic energy when moving can, on its own, explain a number of unintuitive motor behaviors during locomotion (5). And task- and motor-dependent cost functions in optimal feedback control models have proven successful in predicting behavior for other motor systems; for example, those involved in reaching (6). Likewise, the idea that the brain optimizes energy required for neural computations, an important constraint in frameworks like efficient coding and resource rationality, can explain a large range of cognitive and perceptual phenomena (7–10).

Decades of research on fixation selection and gaze behavior in psychology and neuroscience has generated many important insights, including the discovery of reward sensitivity of neural oculomotor circuitry, as well as the conceptual advances of saliency maps, inhibition-of-return, reinforcement learning, and active inference in understanding and modeling gaze behavior (2–4, 11, 12). Perhaps the most general properties of fixation behavior are 1) the center bias, the tendency for the eye to remain mostly near the head orientation/center of the orbit, and return phenomena like 2) spatial inhibition-of-return, the tendency for observers to more often gaze at new locations than recently visited ones, and 3) temporal inhibition-of-return, longer fixation durations preceding a return than preceding a saccade to a new location. Some have speculated about whether biomechanical and energetic efficiency in operating the oculomotor muscles may give rise to the center bias (13–15), but there is no work to our knowledge which actually quantifies how big of an influence this has, versus other factors. Likewise, return phenomena are generally understood to arise from high-level perceptual and cognitive processes (16), for example, the idea that inhibiting return reduces redundancy in information gathered over time (11, 17, 18). Specifically, spatial and temporal inhibition-of-return (IOR) have been classically viewed as consequences of a single mechanism which facilitates foraging (11, 17, 19). However, spatial and temporal IOR do not always accompany one another (20–25), suggesting they are separable and challenging this hypothesis. Furthermore, oculomotor IOR is observed in many other tasks besides search (18, 25, 26) and is an important component of general computational models of fixation and saccade selection (12, 27–30).

In this study, we measured gaze in nine real-world tasks while observers wore a mobile eye tracker and quantified return phenomena and center biases in each. In all tasks, participants made more return than forward saccades and exhibited shorter fixations preceding return than forward saccades; that is, opposite to past laboratory findings taken as support for inhibition-of-return and instead resembling what’s been described as “facilitation of return” (21, 24, 31). This was true even in our visual search/foraging task, “Grocery,” in which participants were tasked with finding and retrieving a specific snack among a large number of different snacks. We observed large eccentricity-dependent differences in fixation probability and duration in all tasks, which on their own quantitatively explained the probability of return as well as fixation durations preceding returns in a simple random sampling model. Our findings suggest that an eccentricity-dependent variable strongly contributes to return phenomena. We speculate that this variable is effort. We propose the hypothesis that energy optimality, specifically minimization of motor costs associated with expected eye and head movements, can parsimoniously account for our observations. Consistent with this hypothesis, the orbital range of motion used in a given task traded off almost linearly with average fixation duration, as if both incur costs in the same space. After controlling for orbital eccentricity, we observed evidence for (temporal) IOR in 1/9 tasks (Lego). Taken together with discrepancies between data and model as well as findings of IOR in previous, head-restrained lab studies, our findings suggest that 1) motor laziness and IOR can both shape fixation selection, 2) their relative contributions depend on task demands and viewing conditions, and 3) under real-world conditions, motor laziness has an outsized influence.

## Results

Each participant performed a subset of nine real-world tasks while their gaze was recorded in head-centered coordinates using the Tobii Pro Glasses 2 mobile eye tracker (accuracy, ±0.62^°^, temporal sampling rate, 50 Hz; see *Materials and Methods* for task and equipment details). Fixations were detected for each recording, yielding estimated scanpaths for each task and participant. We analyzed scanpaths using standard procedures from the literature designed to identify spatial and temporal inhibition-of-return. Next, we simulated synthetic scanpaths by drawing random samples from the probability distribution of fixation locations and the map of average fixation duration— both functions of eye position relative to the (approximate) head orientation. To test whether this random model could capture the phenomena seen in the data, we applied the same analyses to the data and model scanpaths.

### Fixation Position and Duration Displayed Task-Dependent Center Biases.

**Fig. 1.**
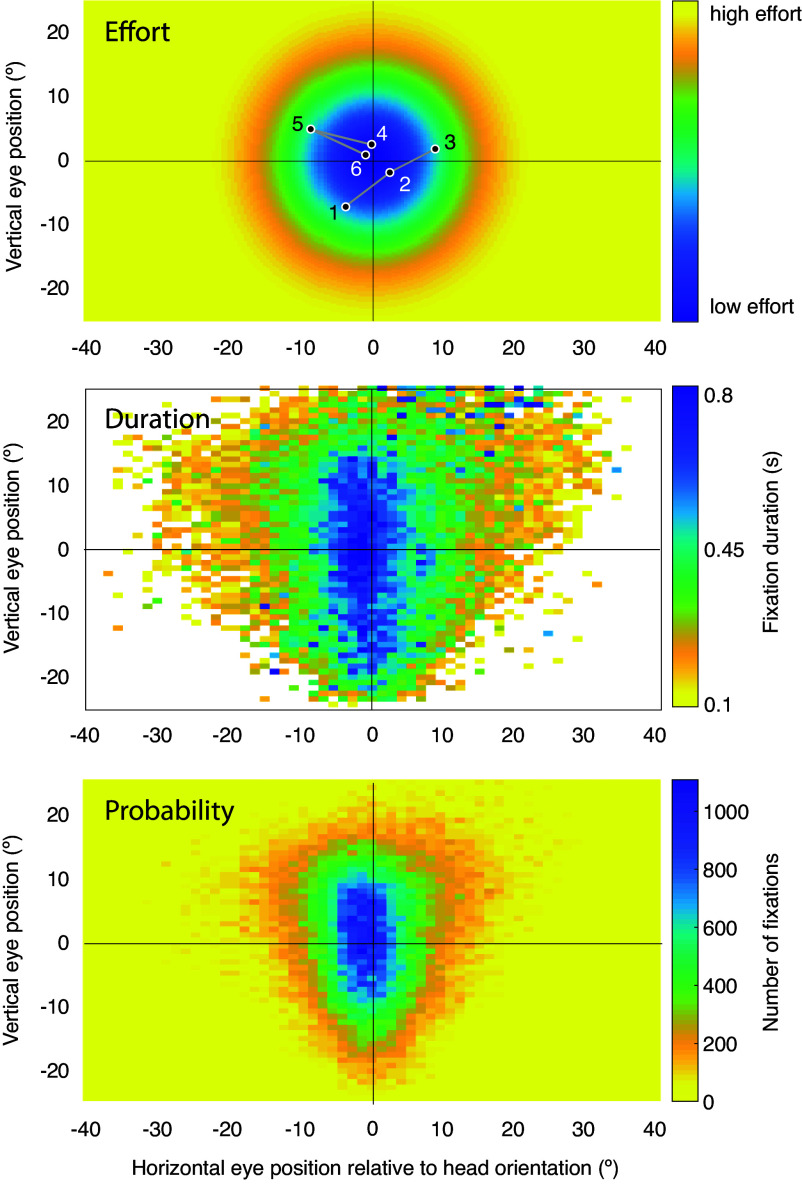
Effort minimization hypothesis of fixation selection. *Top* panel, idealized cartoon depicting effort required to fixate a given orbital position (cross-hairs represent head orientation, which is close to the center of the orbit). We speculate that observers orient their head toward an object of interest, such that when the eye is in the primary position (0,0), it is oriented at or near the object. Fixations 1, 2, and 3 in the cartoon scanpath represent a forward saccade, and 4, 5, and 6 represent a re-fixation. The hypothesis predicts that people will make fewer saccades to locations far from the center of the orbit (validated with data in the *Middle* panel) and that when they do fixate far from the center of the orbit, they will linger there for a shorter amount of time (validated in *Bottom* panel). The *Middle* panel depicts the average fixation duration, and the *Bottom* panel depicts the total number of fixations at a given position relative to the head orientation. Note that the hypothesis predicts that distinct temporal sequences will arise for forward and return saccades—fixation 2 in the sequence is predicted to be longer than fixation 5. Given that more eccentric fixations are rarer and forward sequences take up more orbital range than return sequences, the second fixation in a forward sequence is more likely to be closer to the center of the orbit than the second fixation in a return sequence. To generate a synthetic scanpath, we draw a location sample from the positional distribution in the *Bottom* panel, then look up its duration in the corresponding position in the *Middle* panel, and repeat. Missing values (white) in the *Middle* panel represent position bins for which there were fewer than 10 fixations, such that the mean was dominated by sampling error.

Past studies have identified a center bias in fixation position, where “center” corresponds with the center of the field of view (FOV) in head-fixed free-viewing of images, or the camera/head orientation in head-unrestrained experiments (14, 15, 32–34). Likewise, it is known that more eccentric fixations are maintained for a shorter duration and that more central fixations are maintained for a longer duration (13). We characterized both of these phenomena for each task in our data by estimating 1) the probability distribution of fixation position and 2) a map of average fixation duration, both in head-centered coordinates. To summarize the statistics of fixation position across observers, we divided the FOV into 60 × 60 equal-size bins and summed the total number of fixations across observers and tasks (Fig. 1). Approximately 95% of fixations were within the central 35^°^ of both the horizontal and vertical conditional density passing through the origin. Approximately 50% were within the central 13^°^, vertically, and the central 8^°^, horizontally. Thus, the pooled distribution was considerably peakier than a Gaussian (i.e., leptokurtic). Note that the greater vertical spread was partially due to pooling over task-specific distributions with vertically offset centers (*SI Appendix*, Fig. S1). Also note that many eccentric bins were empty for all tasks and participants, that is, participants very rarely or never fixated in the far periphery. We observed strong center biases in each task, but with large inter-task differences in the center and dispersion of the position distribution (*SI Appendix*, Fig. S1). For fixation duration, we estimated the mean fixation duration in milliseconds within each spatial bin, and for visualization purposes plotted only bins containing more than 10 fixations, for otherwise the mean was dominated by sampling error. For the pooled data (Fig. 1), we observed a similar shape to the fixation position distribution, but more dispersed. The fixations closest to the head orientation averaged 800 ms, whereas the most eccentric fixations averaged 150 ms. There was large inter-task variability in the offset of the duration map (i.e., some tasks had longer fixations overall), as well as in its center and shape (*SI Appendix*, Fig. S2). But generally, fixations were shorter the more eccentric they were.

### Return Saccades Were More Common than Forward Saccades.

**Fig. 2.**
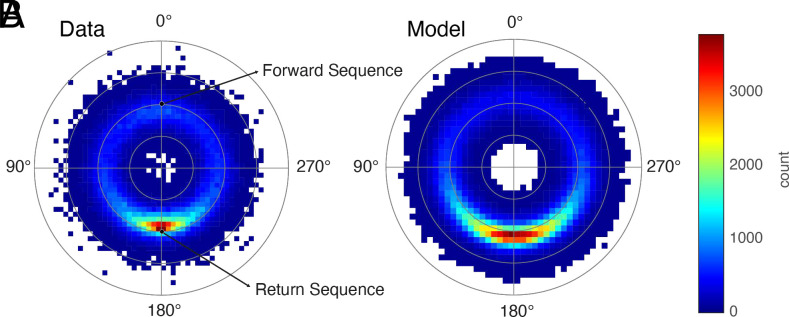
Human and model scanpaths exhibit high rates of re-fixation. (*A*) Relative saccade amplitude and direction joint distribution, i.e., the probability of a saccade having a certain angle and amplitude difference from the preceding one, for data pooled over all tasks and participants. The second ring out from the center corresponds to an amplitude difference of 0^°^. Black circles, idealized exact forward and return saccades, for reference. Therefore, return saccades were the most common type of 3-fixation sequence. The radius axis is in log space. (*B*) Same format as panel *A* but for simulated scanpaths from the model (N is matched in data and model). The ratio of re-fixations to forward saccades in the model was 4.31 (vs. 2.43 in the data). This demonstrates that sampling randomly from the distribution of fixation locations is sufficient to reproduce the spatial facilitation-of-return seen in the data (although overestimating its magnitude).

To assess the degree of spatial inhibition-of-return in our data, we estimated the probability of a saccade having a certain angle and amplitude difference from the preceding one following refs. 18, 24–26. With this joint distribution of relative saccade (log) amplitudes and directions, we measured the spatial IOR ratio: the ratio of return to forward saccades (18), i.e., of the prevalence of saccades within a 90^°^ slice centered on either 180^°^ or 0^°^, respectively, from the previous (see *Materials and Methods* for our operational definition of a saccade). A ratio less than 1 indicates spatial IOR. Surprisingly, we observed a larger number of return saccades relative to forward saccades in all tasks (Fig. 2*A* and *SI Appendix*, Figs. S3 and S8*B*), with the spatial IOR ratio ranging from 1.8 to 3.6 across tasks. This is consistent with spatial “facilitation-of-return.” The most extreme example of this was the Browsing task (ratio = 3.6), in which observers scrolled on their phones (viewing images and reading text), presumably often returning their gaze near the screen center.

### A Random Fixation Selection Model Reproduced High Re-Fixation Rates.

Given that the distribution of fixation positions was focused near the head orientation, it follows that even for a participant who selects fixations randomly and with temporal independence from this distribution, re-fixations should be more probable than forward saccades (assuming that the total amplitudes are roughly matched). This is simply because forward sequences use a larger orbital range of motion than return sequences of the same total amplitude—that is, the average endpoints are more eccentric—and because eccentric fixations are rarer (see Fig. 1*A* for illustration). To test this idea, we simulated scanpaths for each task separately and overall across tasks by drawing random samples from the corresponding distribution of fixation positions. The amount of synthetic and real data was matched. We then estimated the joint distributions of relative saccade directions and amplitudes for the synthetic data, for each task, and computed the spatial IOR ratios as well. The model reproduced the predominance of re-fixations seen in the data in all tasks (Fig. 2*B* and *SI Appendix*, Fig. S3). Overall, the spatial IOR ratio was 1.8 times higher in the model than in the data, and the model did not capture the task dependency in this ratio seen in the data (*SI Appendix*, Fig. S8*B*). This is because, in the model, forward saccades were more equally improbable across tasks, whereas, in the data, their probability varied more across tasks (and was slightly higher overall), which drove task differences in the spatial IOR ratio.

### Fixation Durations Were Longer Preceding Forward than Return Saccades.

**Fig. 3.**
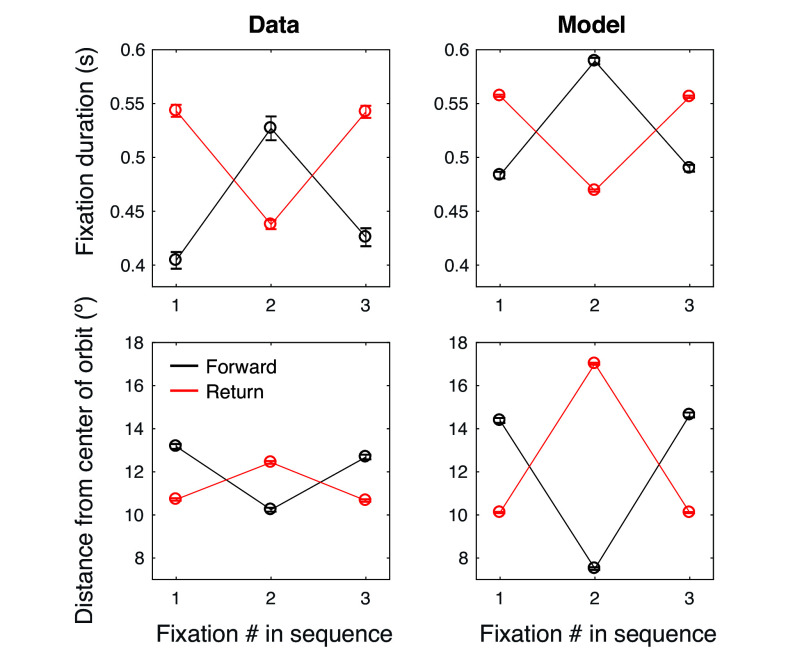
Distinct temporal sequences for forward and return saccade sequences are explained by position of the eye in the orbit, and our model predicts these sequences. *Top Left* panel, avg. fixation duration in seconds for forward and return saccade sequences. Error bar, SEM across fixations (using pre-bootstrapping N). A forward sequence is defined as any two saccades whose angles are within $\pm 25^{{}^{\circ}}$ of one another and whose amplitudes are similar (within 25% of the distribution of relative amplitude distribution for that recording). A return sequence is defined as any two saccades whose angles are within $\pm 25^{{}^{\circ}}$ of 180^°^ from one another and whose amplitudes are similar (within 25% of the distribution of relative amplitude distribution for that recording). *Bottom Left* panel, same as the *Top* panel but the ordinate displays the avg. euclidean distance from the head orientation (Center of the orbit) in degrees of visual angle. *Right* panels, same plots but for model. In both data and model panels, *N* fixations = 16,008 for return sequences and *N* = 1,838 for forward sequences, for each datapoint plotted).

Temporal inhibition-of-return is the tendency for observers to fixate longer preceding a return than forward saccade, when successive saccades are matched in amplitude. Following ref. 18, we defined forward and return saccades as 3-fixation sequences with an absolute relative amplitude of less than the 25% quantile of the distribution (|Δr|< 25%), and a relative angle within 50^°^ (360^°^/7 bins) either precisely forward (0^°^) or backward (±180^°^) (18). The relative amplitude thresholding was done separately for each recording, given that the relative amplitudes varied between tasks (Fig. 3 and *SI Appendix*, Fig. S3). The grand mean fixation duration across all tasks and participants was 520 ms for the second fixation in a forward sequence and 436 ms for the second fixation in a return sequence (*p* < 0.001, one-tailed permutation test, 1,000 permutations). That is, we observed a pattern opposite to temporal IOR, i.e., temporal facilitation-of-return. We found the same pattern (or no evidence for a statistical difference) in all nine tasks (*SI Appendix*, Fig. S4; one-tailed permutation tests, forward > return: *p* = 0.001, 0.011, 0.001, 0.478, 0.038, 0.28, 0.0529, 0.004, 0.265; order of tasks: Browsing, Desk, Driving, Grocery, Lego, Navigation, Restaurant, Pokémon, Walk). No task showed evidence for temporal IOR (i.e., *p* > 0.95, equivalent to the return > forward one-tailed test).

### Forward and Return Saccades Exhibited Distinct Sequences of Fixation Duration, which Were Inversely Proportional to Distance from the Head Orientation.

To better understand our finding of longer fixations preceding a forward versus a return saccade, we looked at the duration of the first and third fixations in each three-fixation sequence. For forward sequences, observers tended to make a shorter fixation followed by a longer fixation, followed by another shorter one. And for return sequences, observers did the opposite. These distinct patterns were observed in the pooled data (Fig. 3) and in each task separately (*SI Appendix*, Fig. S4). Interestingly, when we instead analyzed the distance of these fixations from the head orientation/center of orbit, we found the same patterns but inverted, both for the pooled data and for each task separately (Fig. 3 and *SI Appendix*, Fig. S5). The strikingly strong inverse relationship observed here and in Fig. 1 seems consistent with a mediating relationship, that is, in which the eccentricity of a fixation determines how long it is maintained for.

### Random Fixation Selection Reproduced Fixation Duration and Distance Sequences for Forward and Return Saccades.

To test whether the time-integrated statistics of head-centered fixation position and duration could capture the temporal dependencies, we observed in scanpaths for forward and return sequences, we applied the same analyses used on the data to the synthetic scanpaths generated previously. The model reproduced longer fixations preceding forward than return saccades, as well as distinct sequences of fixation duration and distance from the center of the orbit, which matched the data in their patterns (Fig. 3). The model overestimated fixation durations by a constant offset of around 45 ms. The model also slightly overestimated the difference in duration between forward and return saccades for the second fixation in the sequence, but slightly underestimated the difference for the first and third fixation—more consistent with each curve undergoing an additive offset with the opposite sign, rather than a scaling. Interestingly, this means that the difference between the model and data—that is, what the model does not capture quantitatively in the data—is a temporal-IOR-like effect similar to what past studies have observed, i.e., longer fixations preceding return than forward saccades. This opens the possibility that our data could be described by the sum of two effects, one solely based on distance from the center of the orbit (and captured by our model’s assumptions), and a second, distance-independent effect that is consistent with what has been described previously as temporal IOR, with the distance-dependent effect being considerably larger in our data than the distance-independent/temporal IOR effect. Another possibility, suggested by similar model errors in the distance sequences, is that the second fixation in forward and return sequences are simply further apart (relative to the head orientation) in the model than the data, propagating through to proportional biases in duration.

### Eccentricity-Matched Control Analysis of Fixation Duration Sequences.

To test the idea that a temporal-IOR-like effect may be present in our data, but obscured by a larger eccentricity-dependent effect, we performed a distance-matched control analysis. If this idea is correct, we should observe longer fixations preceding a return than forward saccade, for those fixations which lie within an annulus around the primary position (within some tolerance)—that is, which have the same distance from the head orientation/center of the orbit. Distance matching consisted of finding sequences that were within 2^°^ of the median of the distribution of distances. Distance matching was done for each participant and task separately, after which we pooled the data. We found that, contrary to our prediction, fixation duration was statistically indistinguishable for return and forward sequences, when distance-matched (*SI Appendix*, Fig. S6, permutation test, mean *p*-value = 0.2383, N bootstraps = 100; N permutations per bootstrap = 1,000; only 24% of *p*-values were < 0.05). Given that the sample size after distance-matching was far less, to ensure a fair comparison, we resampled from the raw data using the same sample sizes from the distance-matched data. Specifically, we resampled the data 100 times and performed one permutation test each. The mean *p*-value was 0.0035 and 98% of *p*-values were < 0.05 (N permutations for each test = 1,000). This demonstrates that there was not strong evidence for the presence of temporal IOR in the data overall, even for distance-matched fixations. Next, we looked at each task separately (*SI Appendix*, Fig. S7). Only one task, Lego, showed a statistically significant temporal IOR-like effect (two-tailed permutation test; *p* = 0.001). The Browsing task showed significantly longer fixations for forward than return saccades following distance matching (two-tailed permutation test; *p* = 0.002). There was no evidence of a significant difference for the remaining seven tasks, although the Walk task showed a trend toward an IOR-like effect.

### Fixation Duration and the Orbital Range of Motion Used in a Task Traded Off with Each Other.

**Fig. 4.**
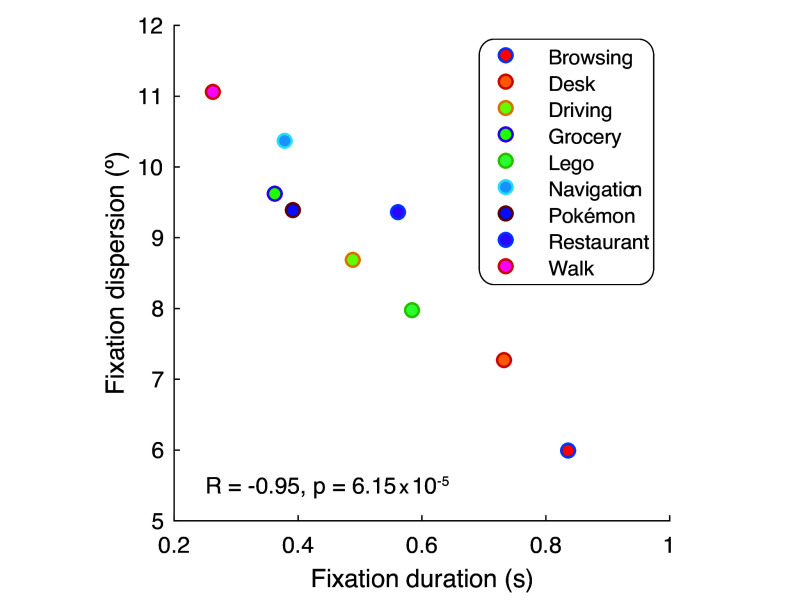
Fixation duration and the orbital range of motion used traded off with each across tasks. Circular symbols, grand mean fixation duration (s) for each task versus fixation dispersion (^°^; measured as the sum of the horizontal and vertical SD of the distribution of fixation locations). Every color is a different task. R and p statistics are from a Pearson’s correlation.

According to our effort minimization hypothesis, the total amount of time the eye is in an eccentric position reflects an underlying oculomotor cost which should be accounted for while also maximizing task-specific rewards. Because humans are fixating observers, this total time should be decomposed into two components—fixation eccentricity and fixation duration—both incurring the same type of cost. Some tasks naturally require a larger orbital range of motion (i.e., more eccentric fixation positions) to gather the same level of reward as others. In such tasks, the eye movement generation circuitry should correspondingly reduce fixation duration, which would help equalize effort expended across many possible tasks. This idea predicts that orbital range and fixation duration should trade off with each other systematically across tasks. To test this, we measured the orbital range of motion used in a given task as the dispersion of fixation positions (sum of the SDs of the horizontal and vertical marginal distributions; *SI Appendix*, Fig. S1). We operationalized fixation duration in this analysis as the grand mean fixation duration across all fixations in a given task. We observed a tight, nearly linear inverse relationship between the two quantities across tasks (Fig. 4; Pearson’s correlation: *r* = −0.95, *p* < 0.001). The Browsing task had the longest fixation duration (838 ms) and the smallest orbital range was used (5.98^°^). The Walk task had the shortest fixation duration (264 ms) and the largest orbital range was used (11.05^°^). This is consistent with the idea that the total amount of time the eye spends in an eccentric position reflects a motor cost that the brain accounts for systematically across tasks with different demands.

### The Influence of Head Movement on Gaze-in-Head Position Did Not Explain Our Findings.

Head-centered gaze measurements have an ambiguity: A change in estimated gaze position can be due to a saccade or to counter-rolling of the eye during a head movement with vestibular ocular reflex (VOR). This creates the possibility that a situation in which—1) the eye starts in the primary position fixated an object X, 2) the observer makes an ocular saccade to an object Y, and 3) then orients their head to object Y (centering the eyes in the orbit again)—will be detected as a return saccade, even though it is not. Despite the fact that our fixation detection method attempts to exclude nonstable fixations (i.e., those during VOR), there can still be head movement between stable fixations, introducing this ambiguity. To control for this, we repeated all our analyses after counter-rotating the gaze-in-head estimates by the momentary head rotation, i.e., transforming them into gaze-in-body coordinates (equivalent to world-locked coordinates when observer translation is zero). Our main findings and conclusions were unchanged (*SI Appendix*, Figs. S9–S13). Spatial IOR ratios and 3-fixation duration sequences for forward and return saccades were similar in head-centered and body-centered coordinates (*SI Appendix*, Fig. S9). Outcomes of statistical hypothesis tests (for detecting temporal IOR) were unchanged. This demonstrates that the possibly problematic scenario described earlier is not as common as the more obvious case of a first saccade followed by a second, return saccade, without substantial head movement in between. This may also be because head movements are much slower than saccades, and the time window of the analysis is just three fixations long. To assess the impact of head movement on the head-centered statistics of fixation position and duration, we simply re-ran our main analyses (in gaze-in-head coordinates), but only for 3-fixation sequences during which head movement was minimal (less than 3^°^/s angular speed, chosen as a threshold to both keep the amount of data high enough to limit sampling error and to minimize head movement). We observed center biases with the same overall shape (*SI Appendix*, Fig. S14 *A* and *B*), demonstrating that head movements do not explain away these effects either. Note that it was not possible to examine return and forward saccades for periods of low head movement in the data, because for low-head-speed epochs with sufficient adjacent fixations, the overall N for some tasks became too small, with sampling error dominating computed metrics. Instead, we used our model to show that when head movement is minimal, we expect to find similar forward/return saccade patterns (*SI Appendix*, Fig. S14 *C*–*F*).

## Discussion

### Overview.

In a wide range of real-world tasks, we found that people tended to keep their eyes in a centered position and that eccentric fixations were much shorter than central ones. These tendencies explain a number of seemingly unrelated phenomena: 1) People return to what they just viewed much more often than they view new locations, 2) fixations preceding returns are shorter than fixations preceding forward gaze shifts, and 3) there are distinct, inverted 3-fixation sequences of durations for return versus forward gaze shifts. We also found that fixation duration and orbital range traded off with each other across tasks. Our results are consistent with the idea that the perceptual-motor system conserves effort, minimizing energetic costs incurred by the specific demands of a task.

### Spatial IOR.

People re-fixate often whether the head is restrained or not. Past work has shown that the presence and extent of spatial IOR varies substantially with task and environment (2, 18, 25, 28, 31). Broadly, tasks that incentivize exploration, like visual search and so-called “free-viewing,” have a higher ratio of forward to return saccades (i.e., they exhibit spatial IOR), whereas many other kinds of tasks exhibit the opposite (spatial facilitation of return) (2, 16, 18, 31). In some tasks, like using a computer or cooking, it is clear a priori that people must re-fixate often to be able to do the task well. That said, most previous studies have used a traditional, head-restrained, static picture viewing laboratory set-up, and so it has been unclear whether spatial IOR is present in real-world, head-unrestrained conditions (2). A recent study by Zhang and colleagues compared the probability of re-fixation in a variety of tasks and viewing conditions, including head-restrained conditions while observers performed free-viewing and visual search tasks, and head-unrestrained conditions (i.e., egocentric video + mobile eye tracking), while observers either made breakfast or searched in a room for items (16). These head-unrestrained data offer a relevant comparison for our present findings. The probability of re-fixation was around 12% on average across their datasets, being highest (30%) for the egocentric task in which observers cooked breakfast and 8% for the egocentric search task. When analyzed in the same way, the average probability of re-fixation across tasks in our data was 5% (5.4% when only analyzing periods when the head was stable, that is, < 3^°^/s angular speed), with the Browsing task having the largest re-fixation probability (13%). We used a 1.5^°^ euclidean distance threshold to define re-fixation events in this analysis, following Zhang et al. Note also, that in our data, re-fixations were the most common type of three-fixation sequence (out of all possible relative angles and amplitudes), and always more common than forward saccades (Zhang et al. did not perform this standard comparison, nor did they analyze temporal IOR, unfortunately). This suggests that in everyday situations, people re-fixate exceedingly often. Even so, our model over-predicted the ratio of forward to return saccades, suggesting that an additional mechanism for generating forward saccades may be needed to explain the data. Spatial IOR may be closely related to such a process (18), which would lend credence to our proposal that both motor laziness and IOR can contribute to fixation selection, with relative strengths depending on task demands and viewing conditions.

### Temporal IOR.

Temporal IOR is a robust phenomenon, observed in a large number of head-restrained studies using a variety of tasks (25, 26). In our study, we observed longer fixations preceding a forward saccade than a re-fixation in nearly all tasks, that is, opposite of the classic pattern seen in past highly controlled lab studies (18, 25, 31). That said, this does not mean temporal IOR was absent in our dataset, if considered in the original sense of a delay in information processing (along with other reductions in performance) before attending or orienting to a previously viewed target, versus other targets (11, 17). Rather, we admit that we cannot determine whether or not such an effect is present in our data, but instead defer to the large body of experimental work on the topic (11, 17). What we can conclude is that there is a seemingly independent, eccentricity-dependent influence on fixation duration in all tasks in our dataset, which is large, and may exist alongside (e.g., sum with) temporal IOR. One piece of evidence in support of this summation account is that our model slightly over-predicted the difference in duration between fixations preceding forward versus return saccades, indicating that a second un-modeled effect with the opposite sign must be added in to reproduce the data. This second effect could be temporal IOR. The second piece of evidence is that when we restricted our analyses to fixations lying in an annulus around the head orientation (i.e., eccentricity-matched), in 1/9 tasks (Lego) we observed longer significantly fixation durations preceding return than forward saccades—that is, consistent with past studies detecting temporal IOR.

### Center Bias.

Center bias is driven, in part, by the tendency to re-center the eyes in their orbits. Observers exhibit a strong tendency to fixate the center of a computer display or picture (14, 15), or near the head orientation in 360^°^ virtual environments (35, 36) and real environments (32–34, 37–39). Past work has shown that there are a number of factors that contribute to the dispersion and center of this center bias, including task demands, environmental layout, FOV size, motor biases, photographer bias, and the tendency to re-center the eyes in the orbit (14, 15). Likewise, in our data, we observed marked differences between tasks in the dispersion and center of the fixation position distribution. Determining the relative contribution of the multiple factors that give rise to these differences is an interesting problem, but beyond the scope of this paper. Instead, we begin from the well-known observation that in head-unrestrained conditions, observers tend to roughly re-center the eyes in the orbit (40) via specific coordinated movements of the eye and head, which are well characterized (40–44). This re-centering behavior must produce some degree of center bias. In the following sections, we will explore the factors that may contribute to this tendency to re-center the eyes in the orbits, including the mobility of the oculomotor muscles, energetic efficiency, and needs of eye-head coordination.

### The Orbital Position–Latency Relation.

Fixation duration scales inversely with the eccentricity of the eye in the orbit, with shorter fixations at more eccentric positions (13, 42, 44–47). Fuller was the first to characterize this relation in humans while also controlling for saccade amplitude, and did so under head-restrained conditions (42). Our head-unrestrained findings are consistent with Fuller’s, with more eccentric fixations maintained for a shorter duration in all tasks. Fuller’s explanation for the orbital position–latency relation is based on the demands of eye-head coordination during gaze shifts (13, 40, 42). Imagine a situation where your left eye is at the left edge of its orbit, but you would like to look at an object even further to the left, out of your field of view. The eye, however, cannot go beyond its edge, so you must initiate a head movement to the left, which will be automatically accompanied by VOR and counter-rolling of the eye to the right. To fixate the target, you must make an ocular saccade to the left. Consequently, in this scenario, the ocular saccade is delayed due to the initial head movement, resulting in an extended period of initial fixation. In fact, this situation corresponds to the longest possible delay. At the other extreme, if you instead wanted to fixate a target near the right edge of the field of view, you would perhaps start with an immediate ocular saccade to the right, then follow it up with a head rotation (and VOR). Fuller found that between these two extremes, the relative onsets of head and eye movement varied lawfully. If gaze shifts of the first type (“ipsiversive”) are rarer than those of the second type (“contraversive”), one would expect to find shorter saccade latencies (and hence fixation durations) on average for more eccentric orbital positions (as both Fuller found and we found in our data). Supporting this eye-head coordination account, individuals with a propensity for more recruitment of head movement during head-unrestrained gaze shifts also exhibit a more pronounced (larger slope) orbital position–latency relation (in head-restrained conditions), and vice versa (41, 42). In summary, the needs of eye-head coordination may partially explain why fixation duration depends on orbital position in our data. However, this does not explain why the eyes tend to end up centered in the orbits following gaze shifts or why contraversive gaze shifts are more prevalent than ipsiversive ones. There appears to be an additional, synergistic reason for the orbital position–latency relation—namely, that the biomechanics and energetic efficiency of the oculomotor muscles facilitates/expedites re-centering of the eye in the orbit. This will be the focus of the next section.

### Oculomotor Biomechanics, Energetic Efficiency, and Re-Centering.

The idea that the brain optimizes energetic costs has proven to be a useful predictive framework for understanding unintuitive motor behaviors in walking (5). Additionally, task- and motor-dependent cost functions in optimal feedback control models have proven successful in predicting behavior for other motor systems such as reaching (6, 48). The same may be true for understanding oculomotor behavior like re-centering and the return phenomena we observed in the present study. Fixations at higher eccentricities require greater muscular force (49, 50), which suggests that they should both be rarer and maintained for a shorter duration if the system is attempting to minimize energetic costs. Furthermore, the oculomotor muscle fibers that are differentially recruited in the maintenance of more eccentric fixations are much less fatigue-resistant than those that keep the eye in the primary position (51). There are also costs associated with the execution of saccades. Re-centering of the eye in the orbit may be facilitated by biomechanical and energetic imbalances between centripetal and centrifugal saccades (those that move the eye from an eccentric to a centered position in the orbit and the opposite, respectively). Centripetal saccades require less force to execute (52), simply due to the fact that passive elastic forces in the oculomotor muscles pull the eye to a centered position at all times, whereas larger, active forces are required to make a centrifugal saccade (53–55). Furthermore, because greater muscle tension is typically required to make a saccade than to fixate, there may be an additional cost related to when to move the eyes versus when to keep them stable (49). In the primary position, the relatively higher energy cost of making any saccade versus continuing to fixate may contribute to longer fixations there. On the other hand, maintaining a 30^°^ fixation (even for 300ms) leads to higher overall (time-integrated) muscle tension than any possible saccade could (49), incentivizing shorter eccentric fixations.

Energy efficiency presumably also explains why contraversive saccades are more common than ipsiversive ones—by definition, ipsiversive saccades use less than half of the orbital range of motion, so will be more eccentric on average (and hence more costly). Re-centering the eyes in the orbit may not only reduce motor costs at the present moment, but also in the near future—specifically it may reduce unnecessary head movements. A head movement is necessary to explore targets beyond the eye’s maximal position in the orbit, but head movements are more costly than eye movements, both in total energy expended and in opportunity costs incurred because of their slow speed of execution (versus ocular saccades) relative to the fast speed of internal processes like perception and planning. Additionally, orbit-eccentric eye orientations may increase the stochasticity of the visuomotor reference frame transformation required to generate actions from those processes, leading to inaccurate or delayed movement execution (56–59). Facing an uncertain future, a centered eye position allows for exploration of a larger range of targets with less costly ocular saccades in either direction, versus more costly head movements (44). Energetic efficiency at the level of neural control of the musculature or more central aspects of fixation/saccade control may also contribute to the re-centering bias (44, 55). Ocular discomfort and fatigue, signals used by the body to prevent injury (60), may also play a role.

### Limitations.

Our dataset, recording device, and analyses have a number of clear limitations. The Tobii Pro Glasses 2 is a mobile eye tracker and as such, when the observer moves more, data quality can be reduced. Likewise, fixation detection becomes more difficult when the observer is moving more, because of the predominance of counter-rolling of the eye during translational and rotational VOR, versus static fixations. Hence, tasks in which observers moved more (e.g., Walk and Navigation) had worse data quality, which may explain larger inter-observer variability in a number of our analyses for these tasks. One limitation of our recording device is that it does not allow us to precisely localize the center of the orbit for each eye (*Materials and Methods*), which some other eye trackers can do. Another limitation is that we could not precisely estimate ocular saccades in world-centered coordinates, as many previous head-fixed lab studies have done. Separating out ocular saccades, counter-rolling of the eye during VOR, smooth pursuit, and static fixations during real-world conditions when an observer is moving is an open research problem and beyond the scope of this work (61). Instead, because we were primarily interested in the properties of scanpaths, stable fixations, and their approximate orbital positions, we focused on identifying stable fixations properly using well-accepted methodology (Velocity-Threshold Identification [I-VT]; see *Materials and Methods* for details) and we did this estimation in head-centered coordinates in order to assess approximate orbital position (16).

Different definitions of return saccades have been used in some past studies (16, 22, 24)—for example, looking back further than one saccade into the past. These are also valid and interesting to study. We focused on 1-back return saccades in this paper for a few reasons: 1) for simplicity, as this is a first presentation of our ideas and data; 2) we specifically aimed to make sense of 3-fixation forward and return sequences (4-fixation forward sequences are virtually absent in our data, for example); and 3) following relevant past work (18), to facilitate comparison. That said, analyzing 2-back, 3-back, and n-back return saccades, and seeing how these relate to gaze-in-head position, would be a great topic for future work. Relatedly, return saccades have been defined either based on the euclidean distance of their landing position from a prior fixation (16) or on their angle and amplitude difference from a preceding saccade (18, 24–26). The two methods are similar in that they both specify a qualifying spatial region for a return fixation, but the regions have different shapes (the former is a circle and the latter is an annulus sector). We found high rates of return saccades across tasks using either definition (see *Discussion* “Spatial IOR” for euclidean-distance-based analysis). It would also be interesting to analyze the probability of re-fixation on specific world-locked objects/positions as well as the average amount of time between re-fixations on the same object/position (i.e., the inter-return interval). One could then compare re-fixation rates for objects that appear across different tasks, for example. Given that some of our tasks included more observer translation than others (and objects could therefore become nonvisible as observers moved), such analyses over the relevant time epochs were obviated by our chosen head-centered coordinate system and short-timescale approach (using 3-fixation epochs).

While we attempted to include tasks that reflect a wide range of common everyday activities, there are undoubtedly activities our findings do not cover or generalize to. There may be real-world activities in which forward saccades are more beneficial than return saccades, for example, leading to classical signatures of IOR, which match past lab studies. Such a finding could help identify the specific contextual factors that explain discrepancies between real-world and lab settings in return/forward saccade probabilities (and latencies).

### Open Questions.

We are unable to explain why past lab studies observed longer fixations preceding return than forward saccades, whereas we observed the opposite. This apparent lack of temporal IOR could be a result of differences in the type of tasks used and/or the ability to move the head and body freely. For example, many (but not all) past studies identifying temporal IOR used free-viewing or visual search tasks, which incentivize exploration of the visual image, a demand which may inhibit the natural tendency to re-center the eye in the orbit. So this account predicts that these past studies will show a smaller proportion of re-centering saccades (versus other types of saccades) than our study. Additionally, this past work implies that temporal IOR was present in our data but obscured by a stronger eccentricity-dependent effect of fixation duration. Another explanation is that the orbital position–latency relation is overall stronger in head-unrestrained versus head-restrained conditions because it has a greater utility in active eye-head coordination. That said, it is known that the orbital position–latency relation still holds even in head-restrained conditions (13) and furthermore, the neck muscles show activity related to eye-head coordination and saccade planning even when observers are head-restrained (62). So this raises a worthwhile empirical question concerning the relative contributions of two putative components in different task contexts: an eccentricity-dependent one (the orbital position–latency relation) and an eccentricity-independent one (temporal IOR). Another interesting avenue for future work is quantitatively modeling the energy and opportunity costs involved in generating head and eye movements. If such a model is able to predict re-centering as an optimal strategy, that would be useful. Furthermore, a number of models of fixation selection and scanpath generation depend on spatial-IOR-like mechanisms (12, 27–30, 63), some of which are able to reproduce task differences in gaze behavior. It will be interesting to re-consider those mechanisms in the context of our findings, especially as such models may be extended to work with egocentric video.

In some of our tasks, e.g., Navigation and Driving, parts of the visual stimulus/environment changed over time as the observer moved. One might ask whether return saccades, and hence IOR, is even defined in such cases, given that a previously fixated object might be un-viewable at the next moment. What matters for our operational definition of a return saccade is that the area in space where the observer is harvesting information at different moments in time is the same, not that the exact contents within that area are identical. A similar definition has been used in a past study examining return saccades in egocentric, unrestrained viewing conditions (16). For example, when driving in a car around a bend, it is known that observers tend to fixate at a certain part of the road ahead to effectively steer (64). In such a case, over time, the visual input changes, but also conveys textural, motion, and depth information that is similar at different moments in time (64, 65), and drivers tend to gaze at the same body-relative positions over time (same when they check the speedometer or GPS, which are also in fixed body-relative positions). In fact, in most natural situations, the sensory environment contains redundancies even as the information content changes. A different, but complementary example is scrolling through content on a smartphone. In this case, the exact text or images on the screen change over time as one scrolls, while the information content also changes, but the visual region near the center of the screen where you harvest most information is small and relatively fixed over time. Therefore, a person in a natural situation might check the clock at the top of their phone’s screen, then look back to the center of the screen where they are browsing, look up from their phone for a moment, then back down to the center, and so on. These can be considered return saccades in our definition, because they return to the same area where information is being harvested. This definition is also supported by our data—observers displayed strong regularities in where they most often looked relative to their bodies (*SI Appendix*, Fig. S10) and in the prevalence of return saccades in body-centered coordinates (*SI Appendix*, Fig. S12).

Another important question is how the fixated content interacts with return/forward saccade probabilities and durations. For example, does the spatial or spatiotemporal retinal image spectrum differ for return and forward saccades? If so, how does that affect their probability of occurrence or the maintenance of fixation? In this study, we were interested in low-level motor constraints on fixation selection, but low-level visual factors also influence what someone chooses to look at. These include the benefits of certain saccades for low-level visual information processing, e.g., spatiotemporal reformatting (66), and the association between saliency and low-level image features (spatial frequency, luminance, contract, color) (2, 24). For example, a past study found that return saccades are directed to more salient locations in natural images versus other saccades (24). Future work can analyze egocentric videos—looking at the spectral content around fixation—to probe these questions. Another interesting question for future research is whether people tend to point their heads at objects that they will subsequently re-fixate multiple times, and how head–eye coordination strategies based on energy optimality interact with task demands. Labeling and tracking fixated objects in egocentric videos (like the ones in our dataset) with computer vision methods provides an avenue to begin answering this question.

We observed substantial inter-task variability in the center and dispersion of the fixation position distributions. For example, in some tasks, the center (i.e., the most probable fixated location) was around 5^°^ from the head orientation (*SI Appendix*, Figs. S1 and S2). Such differences are interesting, although we can only speculate about their causes (as we did not analyze the world videos or fixated content). We view the most probable fixation position as a compromise between task demands (which constrain the head orientation and thereby bias the chosen gaze-in-head position) and numerous other factors, including those related to gaze-in-head position itself (i.e., motor laziness, eye strain). One of the strengths of our modeling approach is that we can simulate task-specific fixations based on these diverse distributions, and still successfully predict strong regularities in return and forward saccade properties shared between tasks. That is, our findings do not depend on the center bias having a stereotyped shape—being centered at the head orientation exactly, for example. We speculate that in many real-world situations, the brain will “override” what is most comfortable or energy efficient—e.g., by maintaining a very eccentric fixation—if a task requires it, because other demands/rewards are more critical. For example, while driving, at certain moments there may be only a small range of safe head positions (given the slow speed of head movements relative to speed of action on the road), so in such cases, it might be optimal to make a very eccentric fixation even though it is uncomfortable or inefficient. That’s an extreme case, but in other tasks, it may be optimal (in a reward-maximizing sense) for task performance to keep the eyes pointed at an object 5^°^ to 15^°^ from the head orientation, for example. Such biases are not inconsistent per se with energy optimality, but future work modeling the energetic cost of different eye positions and saccades relative to the possible cost of alternative movements (e.g., head, body re-orientations that achieve a similar aim) may be able to make some of these speculations more quantitative. Additionally, we found that the orbital range of motion participants used in a specific task (i.e., the fixation dispersion) was closely associated with how long they fixated on average in that task (Fig. 4). This emphasizes how varying tasks can help unveil both the adaptability and efficiency of gaze behavior.

Visual search is the canonical task associated with IOR, where it was proposed as a foraging facilitator (11, 17). However, in our naturalistic, head- and body-free visual search task (“Grocery”), which was also similar to a foraging task, we observed spatial and temporal IOR signatures opposite to what have been seen in most lab studies of visual search. Furthermore, we did not observe even residual evidence for IOR in our search task after controlling for the influence of orbital eccentricity. Encouragingly, Zhang et al. found a similar result in another naturalistic visual search task, in which participants were tasked with finding a specific object in a room while wearing a mobile eye tracker (16). They found return fixation probabilities of 8%, even higher than what we found. One possible explanation is that observers override ocular comfort and energetic efficiency disproportionately during lab studies of visual search, leading to unnatural sequences of eye movements that are tightly tailored to task demands. This does not invalidate IOR as a search mechanism, but instead suggests that it may be part of a larger repertoire of natural eye/head movement strategies that are functionally specific.

### Conclusion.

In this paper, we argue that the tendency to re-center the eyes in the orbit gives rise to a number of interesting properties of real-world scanpaths, including the predominance of return saccades (16). The re-centering tendency itself seems to be due to a combination of multiple, synergistic factors: 1) neural and muscular energetic efficiency, 2) the limits and needs of eye-head coordination, and 3) higher-level perceptual, attentive, planning, and inferential processes that attempt to identify and orient to future targets. We can only speculate about the importance of each factor. Our current findings cannot speak to the relative contributions of each and how these vary with task demands and environment. The development of models which quantify the contributions of these factors is critical to understanding fixation selection. Much of the literature on inhibition-of-return and re-centering has treated peripheral muscular constraints as an uninteresting null hypothesis, so it is unclear how much of seemingly high-level eye movement behaviors can be explained by low-level factors. Given that it is far more tractable to model the biomechanics and energetics of the oculomotor muscles than to model perception and cognition, we argue that the former should be done first. As a simplest next step, existing biophysical models of the eye plant (50, 52) can be re-configured as scanpath models by combining them with probabilistic sampling and optimizers that minimize physical energy (which some models already output). Some work already exists in trying to approximate user effort with oculomotor energy computed by eye plant models (67). In general, models of gaze behavior at many levels of abstraction can incorporate oculomotor costs by considering fixation eccentricity as a proxy for effort (for head-fixed observers) and treating this as a (negative) reward, including active inference models of gaze behavior (3) and reinforcement learning models (4). Deep learning models may have already learned this cost implicitly, hence why some machine learning researchers (correctly) try to factor out the predictiveness of the center bias on its own when evaluating saliency/gaze prediction deep learning models (68). Regarding IOR in everyday contexts, considered in the context of past studies, our findings suggest that IOR may be present in certain everyday tasks but weak compared to other factors influencing fixation selection (like energy efficiency and comfort).

## Materials and Methods

### Participants.

Thirty participants (ten internal employees and twenty external participants) took part in the study. All participants had normal or corrected-to-normal vision. The self-exclusion criterion for participants was anyone outside of an age range of 18 to 65 y old. Gender and age demographics were not recorded, so we cannot account for any age or gender-related differences between participants. Data were collected internally at Reality Labs, Meta Platforms Inc., in accord with the Declaration of Helsinki, and the study went through the organization’s research ethics review process (including independent Legal, Operations, Safety, and Program Manager approvals). All participants provided informed consent for their participation in the study.

### Data Collection.

Each participant performed a variable subset of nine possible tasks, which ranged from approximately 5 to 20 min in duration. Participants wore the eye tracker during task performance, and in some cases operated the device themselves and in other cases were assisted by a research assistant. Tasks were selected to span a range of normal day-to-day activities and because these activities might benefit from augmentation. Some participants only performed one task, and others performed up to 6 different tasks, obviating systematic within-participants comparisons. The task descriptions and number of participants who performed each were as follows:

1. Driving (*N* = 7). Participants drove to or from work.

2. Browsing (*N* = 30). Participants browsed the web with their mobile phone while seated.

3. Lego (*N* = 30). Participants built a Lego set using instructions while seated at a table.

4. Walk (*N* = 9). Participants walked outside on a forest path.

5. Desk (*N* = 24). Participants worked on their computer at their desk.

6. Pokémon (*N* = 18) Participants played an augmented reality mobile game (Pokémon GO) on their phone outside a building (69).

7. Restaurant (*N* = 19). Participants ordered a meal on a tablet and spoke to a waiter as well. Note that this task was “acted” in a conference room with a confederate acting as a “waiter,” and an electronic tablet for a “menu.”

8. Grocery (*N* = 20). Participants were tasked with visually locating and then retrieving a pre-specified item (a snack) in a kitchen, by searching through shelves and drawers full of many different snacks with varying visual appearances. This was specifically designed to be a naturalistic visual search task. We called it “Grocery” because it most closely simulates a grocery shopping experience.

9. Navigation (*N* = 10). Participants were tasked with finding a specific conference room in a research building and navigating there using a digital map on the wall and their phone.

Note that for each task (except Driving), each participant conducted the task in a very similar environment.

### Gaze Measurement.

Gaze was recorded using a Tobii Pro Glasses 2 mobile head-mounted eye tracker. The eye tracker contains two eye cameras and eight infrared illuminators per eye, and uses a combination of corneal reflection, dark pupil, and stereo geometry to achieve gaze measurements at 50 Hz with an angular error of ±0.62^°^ on average (70). The Tobii firmware also accounts for slippage using a 3D geometric eye model. The trackable gaze range of the eye camera was 80^°^ horizontally by 50^°^ vertically. Gaze data were stored and processed on-device by Tobii’s proprietary firmware. We will refer to the coordinate system of the gaze data as “gaze-in-head” or “head-centered”—as the gaze measurements are always relative to the orientation of the head. Specifically, the 3D origin of the gaze-in-head data was internally computed by the Tobii Pro firmware based on the estimated eye locations relative to the eye cameras at the time of calibration. This origin is located at the midpoint of the inter-ocular axis on the surface of the Tobii headset. Therefore, gaze measurements from the two eyes were transformed such that they were with respect to a hypothetical cyclopean observer.

Participants followed a single-point calibration method supplied by the manufacturer (71). During this calibration, participants held a card with a target at arm’s length and approximately at the interocular axis elevation. The system used this calibration sequence to determine the best fit eye model and where it was relative to the cyclopean origin based on their proprietary internal algorithm. There is a possibility of an upward vertical bias in the gaze data, if certain participants accidentally held the calibration card below their interocular axis. That said, we did not observe fixation locations across tasks that were systematically above those measured in past studies with similar mobile eye tracking methods (32, 33, 37–39), so we do not suspect such a bias is significant in our dataset.

We observed a larger spread vertically than horizontally of gaze-in-head positions in some tasks but not others (*SI Appendix*, Fig. S1). We do not believe that this was an artifact caused by device slippage given that 1) the eye tracker was affixed well to the head, 2) we do not see a consistent vertical spread bias across tasks or observers, and 3) past studies have observed similar task-dependent anisotropies (see ref. 39, their figure 3C - “Make a Sandwich” task vs. others; also see ref. 33).

### Fixation Detection.

To detect gaze fixations, we used Tobii’s I-VT fixation detection method (implemented in the Tobii Pro Lab software), which employs a velocity threshold of 30^°^/s. This method attempts to exclude smooth pursuit, VOR, and ocular following eye movements, which are also types of fixations, but cause the gaze-in-head estimates to shift over time. Instead, the fixation detection method attempts to detect only “stable fixations,” for which head movement is minimal. We used this method to be conservative, given concerns about fixation estimate quality during head movement there is a dearth of methods for detecting fixations during VOR/smooth pursuit for mobile eye trackers (61). The I-VT method also merges fixations that are within a short interval of time, and accounts for measurement noise in the gaze estimates inherent to the Tobii Pro Glasses 2 mobile eye tracker.

Mobile eye trackers produce gaze and fixation measurements that are similar in accuracy from those from a standard EyeLink device (SR Research, Ottawa, Ontario), particularly when analyzed on average across measurements and participants (61). Furthermore, saccade amplitudes and fixation durations we observed were within the range of what others have obtained with more precise devices. Therefore, we were confident in the fidelity of the gaze measurements.

### Saccade Estimation.

We estimated saccades simply as the vector of displacement between two subsequent estimated fixation points in gaze-in-head coordinates. As a result, what we call a “saccade” in our head-centered analyses may in some cases actually represent a gaze shift (i.e., some combination of an ocular saccade and counter-rolling of the eye during head movement/VOR). Note that this was not a large concern because we were primarily interested in the properties of fixations and scanpaths, not ocular saccades, and in particular the orbital position of the eye during fixation. That said, to determine whether these ambiguities could be having a large influence on our primary results, we analyzed saccade directions and amplitudes using our data and found that were comparable to known metrics in the literature (*SI Appendix*). A few examples of return and forward saccades from the data, in head-centered coordinates, are displayed in *SI Appendix*, Fig. S15.

### Model.

We used a random sampling model to generate synthetic scanpaths, which consisted of a sequence of N fixations, each comprising 1) a horizontal position in degrees relative to the head orientation, 2) a vertical position in degrees relative to the head orientation, and 3) a duration in seconds. To generate a synthetic scanpath, N random independent samples were drawn from the empirical probability distribution of fixation locations, where N is equal to the number of datapoints in the empirical distribution, generating horizontal and vertical positions for each synthetic fixation. Next, the duration of a synthetic fixation was determined by simply looking up the duration in the corresponding horizontal and vertical position in the empirical map of average fixation durations. The empirical probability distribution of fixation locations and the duration map shared the spatial sampling rate, allowing for this “look-up table” approach. We synthesized scanpaths in this manner for each task separately (based on the task-specific distributions) and for the pooled data across all tasks (based on the pooled distributions).

The model has two primary assumptions: 1) the duration of a fixation is only determined by its position relative to the head orientation (and the duration statistics at that position), and 2) each fixation is independent of the specific content of the environment and the past history of fixations. Regarding point (1), the probability distribution of fixation duration at each location in the spatial map was summarized with the mean, so the model also ignores this variability. This approach also specifically ignores any other factors that are known to shape saccade selection and generation. Note that the model is simple by design. It is not meant to reproduce many aspects of fixation and saccade behavior, but rather to probe how far one can get in explaining temporal dependencies in fixation position and duration without explicitly specifying any cognitive or perceptual processes, and by assuming a random, temporally independent process that only depends on orbital position. In this way, what is interesting is, quantitatively, how much the model can and cannot explain—which gives clues about the relative contributions of the processes that shape gaze behavior.

### Data Equalization.

Each task had a different N due to the variable set of participants performing it. Therefore, for all pooled analyses, we used bootstrapping to equalize N across tasks prior to pooling. Bootstrapping was always applied to the metric of interest, rather than to the input data, given that task-specific thresholds were often used in our analyses. For Figs. 1 and 2, for example, we used bootstrapping to re-sample extra fixation position-duration pairs for each task separately (using just enough iterations to match the task with the highest N), then pooled across tasks. Data were always equalized between data and model as well by either simulating more fixations with the model or bootstrapping metrics derived from the model fixations.

## Supplementary Material

Appendix 01 (PDF)

## Data Availability

Code and anonymized data have been deposited in an Open Science Foundation repository (72).

## References

[1] J. Najemnik, W. S. Geisler, Eye movement statistics in humans are consistent with an optimal search strategy. J. vision **8**, 4.1–414 (2008). 10.1167/8.3.4 PMC2868380 18484810

[2] B. W. Tatler, M. M. Hayhoe, M. F. Land, D. H. Ballard, Eye guidance in natural vision: Reinterpreting salience. J. Vis. **11**, 5 (2011). 10.1167/11.5.5 PMC3134223 21622729

[3] M. B. Mirza, R. A. Adams, K. J. Friston, T. Parr, Introducing a Bayesian model of selective attention based on active inference. Sci. Rep. **9** (2019). 10.1038/s41598-019-50138-8 PMC6763492 31558746

[4] A. Radulescu, B. van Opheusden, F. Callaway, T. L. Griffiths, J. M. Hillis, Modeling human eye movements during immersive visual search. bioRxiv (2022). https://www.biorxiv.org/content/10.1101/2022.12.01.518717v1 (Accessed 1 February 2023). 10.1162/OPMI.a.322 PMC13053022 41948075

[5] G. L. Brown, N. Seethapathi, M. Srinivasan, A unified energy-optimality criterion predicts human navigation paths and speeds. Proc. Natl. Acad. Sci. U.S.A. **118** (2020). 10.1073/pnas.2020327118 PMC8307777 34266945

[6] S. H. Scott, Optimal feedback control and the neural basis of volitional motor control. Nat. Rev. Neurosci. **5**, 532–546 (2004). 15208695 10.1038/nrn1427

[7] X. Wei, A. A. Stocker, Lawful relation between perceptual bias and discriminability. Proc. Natl. Acad. Sci. U.S.A. **114**, 10244–10249 (2017). 28874578 10.1073/pnas.1619153114 PMC5617240

[8] J. Gervain, M. N. Geffen, Efficient neural coding in auditory and speech perception. Trends Neurosci. **42**, 56–65 (2019). 30297085 10.1016/j.tins.2018.09.004 PMC6542557

[9] R. Polanía, M. Woodford, C. C. Ruff, Efficient coding of subjective value. Nat. Neurosci. **22**, 134–142 (2018). 30559477 10.1038/s41593-018-0292-0 PMC6314450

[10] A. Prat-Carrabin, M. Woodford, Efficient coding of numbers explains decision bias and noise. Nat. Hum. Behav. **6**, 1142–1152 (2022). 35637295 10.1038/s41562-022-01352-4

[11] R. M. Klein, Inhibition of return. Trend. Cognit. Sci. **4**, 138–147 (2000). 10.1016/s1364-6613(00)01452-2 10740278

[12] L. Itti, C. Koch, Computational modelling of visual attention. Nat. Rev. Neurosci. **2**, 194–203 (2001). 11256080 10.1038/35058500

[13] J. H. Fuller, Comparison of Head Movement Strategies among Mammals in The Head-Neck Sensory Motor System (Oxford University Press, 1992).

[14] B. W. Tatler, The central fixation bias in scene viewing: Selecting an optimal viewing position independently of motor biases and image feature distributions. J. Vis. **7**, 4 (2007). 10.1167/7.14.4 18217799

[15] P. H. Tseng, R. Carmi, I. G. M. Cameron, D. P. Munoz, L. Itti, Quantifying center bias of observers in free viewing of dynamic natural scenes. J. Vis. **9**, 4 (2009). 19761319 10.1167/9.7.4

[16] M. Zhang , Look twice: A generalist computational model predicts return fixations across tasks and species. PLoS Comput. Biol. **18** (2021). 10.1371/journal.pcbi.1010654 PMC9681066 36413523

[17] R. M. Klein, W. J. MacInnes, Inhibition of return is a foraging facilitator in visual search. Psychol. Sci. **10**, 346–352 (1999).

[18] P. M. Bays, M. Husain, Active inhibition and memory promote exploration and search of natural scenes. J. Vis. **12**, 8 (2012). 10.1167/12.8.8 PMC3587005 22895881

[19] M. I. Posner , Components of visual orienting. Atten. Performance X: Control Lang. Process. **32**, 531–556 (1984).

[20] I. T. Hooge, E. A. Over, R. J. van Wezel, M. A. Frens, Inhibition of return is not a foraging facilitator in saccadic search and free viewing. Vis. Res. **45**, 1901–1908 (2005). 15797779 10.1016/j.visres.2005.01.030

[21] T. J. Smith, J. M. Henderson, Facilitation of return during scene viewing. Visual Cognit. **17**, 1083–1108 (2009).

[22] T. J. Smith, J. M. Henderson, Looking back at Waldo: Oculomotor inhibition of return does not prevent return fixations. J. Vision **11**, 3–3 (2011). 10.1167/11.1.3 21205873

[23] T. J. Smith, J. M. Henderson, Does oculomotor inhibition of return influence fixation probability during scene search? Atten. Percep. Psychophys. **73**, 2384–2398 (2011). 10.3758/s13414-011-0191-x 21837543

[24] N. Wilming, S. Harst, N. Schmidt, P. König, Saccadic momentum and facilitation of return saccades contribute to an optimal foraging strategy. PLoS Comput. Biol. **9**, 1–13 (2013). 10.1371/journal.pcbi.1002871 PMC3547797 23341766

[25] S. G. Luke, T. J. Smith, J. Schmidt, J. M. Henderson, Dissociating temporal inhibition of return and saccadic momentum across multiple eye-movement tasks. J. Vision **14**, 9 (2014). 10.1167/14.14.9 25527147

[26] M. Nadezhda, K. Dovbnyuk, L. Merzon, W. J. MacInnes, Between the scenes. Exp. Psychol. **69**, 185–195, PMID: 36305454 (2022). 36305454 10.1027/1618-3169/a000556 PMC9730397

[27] R. Engbert, H. A. Trukenbrod, S. Barthelmé, F. A. Wichmann, Spatial statistics and attentional dynamics in scene viewing. J. Vision **15**, 14–14 (2015). 10.1167/15.1.14 25589298

[28] L. O. Rothkegel, H. A. Trukenbrod, H. H. Schütt, F. A. Wichmann, R. Engbert, Influence of initial fixation position in scene viewing. Vision Res. **129**, 33–49 (2016). 27771330 10.1016/j.visres.2016.09.012

[29] L. Schwetlick, L. O. M. Rothkegel, H. A. Trukenbrod, R. Engbert, Modeling the effects of perisaccadic attention on gaze statistics during scene viewing. Commun. Biol. **3** (2020). 10.1038/s42003-020-01429-8 PMC7708631 33262536

[30] L. Schwetlick, D. Backhaus, R. Engbert, A dynamical scan-path model for task-dependence during scene viewing. Psychol. Rev. **130** (2023). 10.1037/rev0000379 36190753

[31] S. G. Luke, J. Schmidt, J. M. Henderson, Temporal oculomotor inhibition of return and spatial facilitation of return in a visual encoding task. Front. Psychol. **4** (2013). 10.3389/fpsyg.2013.00400 PMC3698447 23847574

[32] T. Foulsham, E. Walker, A. Kingstone, The where, what and when of gaze allocation in the lab and the natural environment. Vision Res. **51**, 1920–1931 (2011). 21784095 10.1016/j.visres.2011.07.002

[33] F. Ioannidou, F. Hermens, T. L. Hodgson, The central bias in day-to-day viewing. J. Eye Move. Res. **9** (2016).

[34] D. Backhaus, R. Engbert, L. O. M. Rothkegel, H. A. Trukenbrod, Task-dependence in scene perception: Head unrestrained viewing using mobile eye-tracking. J. Vision **20** (2019). 10.1167/jov.20.5.3 PMC7409614 32392286

[35] J. Hadnett-Hunter, G. Nicolaou, E. O’Neill, M. Proulx, The effect of task on visual attention in interactive virtual environments. ACM Trans. Appl. Percept. **16** (2019).

[36] Z. Hu, A. Bulling, S. Li, G. Wang, Fixationnet: Forecasting eye fixations in task-oriented virtual environments. IEEE Trans. Visual. Comput. Graphics **27**, 2681–2690 (2021). 10.1109/TVCG.2021.3067779 33750707

[37] Y. Li, A. Fathi, J. M. Rehg, “Learning to predict gaze in egocentric video” in *2013 IEEE International Conference on Computer Vision* (IEEE, Sydney, NSW, Australia, 2013), pp. 3216–3223.

[38] K. S. Kretch, K. E. Adolph, Active vision in passive locomotion: Real-world free viewing in infants and adults. Dev. Sci. **18**, 736–750 (2015). 25438618 10.1111/desc.12251 PMC4447601

[39] W. W. Sprague, E. A. Cooper, I. Tošić, M. S. Banks, Stereopsis is adaptive for the natural environment. Sci. Adv. **1**, e1400254 (2015). 26207262 10.1126/sciadv.1400254 PMC4507831

[40] M. F. Land, Predictable eye-head coordination during driving. Nature **359**, 318–320 (1992). 1406934 10.1038/359318a0

[41] J. H. Fuller, Head movement propensity. Exp. Brain Res. **92**, 152–164 (1992). 1486950 10.1007/BF00230391

[42] J. H. Fuller, Eye position and target amplitude effects on human visual saccadic latencies. Exp. Brain Res. **109**, 457–466 (1996). 8817276 10.1007/BF00229630

[43] D. B. Tweed, Visual-motor optimization in binocular control. Vision Res. **37**, 1939–1951 (1997). 9274779 10.1016/s0042-6989(97)00002-3

[44] M. Paré, D. P. Munoz, Expression of a re-centering bias in saccade regulation by superior colliculus neurons. Exp. Brain Res. **137**, 354–368 (2001). 11355382 10.1007/s002210000647

[45] A. J. van Opstal, K. Hepp, Y. Suzuki, V. Henn, Influence of eye position on activity in monkey superior colliculus. J. Neurophysiol. **74**, 1593–610 (1995). 8989396 10.1152/jn.1995.74.4.1593

[46] D. Zambarbieri, G. Beltrami, M. Versino, Saccade latency toward auditory targets depends on the relative position of the sound source with respect to the eyes. Vision Res. **35**, 3305–3312 (1995). 8560801 10.1016/0042-6989(95)00065-m

[47] R. M. Krebs, M. A. Schoenfeld, C. N. Boehler, A. W. Song, M. G. Woldorff, The saccadic re-centering bias is associated with activity changes in the human superior colliculus. Front. Hum. Neurosci. **4** (2010). 10.3389/fnhum.2010.00193 PMC2987555 21103010

[48] R. Shadmehr, M. A. Smith, J. W. Krakauer, Error correction, sensory prediction, and adaptation in motor control. Annu. Rev. Neurosci. **33**, 89–108 (2010). 20367317 10.1146/annurev-neuro-060909-153135

[49] C. Collins, D. M. O’Meara, A. B. Scott, Muscle tension during unrestrained human eye movements. J. Physiol. **245** (1975). 10.1113/jphysiol.1975.sp010850 PMC1330791 1142165

[50] O. Komogortsev, Ph.D. thesis (Kent State University) (2007).

[51] R. J. Leigh, D. S. Zee, The Neurology of Eye Movements, Contemporary Neurology Series (Oxford University Press, Oxford, 2015), (ed. 5, p. 90).

[52] A. R. Koene, C. J. Erkelens, Cause of kinematic differences during centrifugal and centripetal saccades. Vision Res. **42**, 1797–1808 (2002). 12127111 10.1016/s0042-6989(02)00110-4

[53] D. A. Robinson, Control of eye movements. *Compreh. Physiol.* 1275–1320 (1981).

[54] D. Pelisson, C. Prablanc, Kinematics of centrifugal and centripetal saccadic eye movements in man. Vision Res. **28**, 87–94 (1988). 3414002

[55] J. Tagu, K. Doré-Mazars, J. Vergne, C. Lemoine-Lardennois, D. Vergilino-Perez, Recentering bias for temporal saccades only: Evidence from binocular recordings of eye movements. J. Vision **18**, 10 (2018). 10.1167/18.1.10 29356814

[56] J. K. Burns, G. Blohm, Multi-sensory weights depend on contextual noise in reference frame transformations. Front. Hum. Neurosci. **4** (2010). 10.3389/fnhum.2010.00221 PMC3002464 21165177

[57] J. K. Burns, J. Y. Nashed, G. Blohm, Head roll influences perceived hand position. J. Vision **11**, 9 (2011). 10.1167/11.9.3 21824979

[58] H. Alikhanian, S. R. de Carvalho, G. Blohm, Quantifying effects of stochasticity in reference frame transformations on posterior distributions. Front. Comput. Neurosci. **9** (2015). 10.3389/fncom.2015.00082 PMC4490245 26190998

[59] T. S. Murdison, D. I. Standage, P. Lefèvre, G. Blohm, Effector-dependent stochastic reference frame transformations alter decision-making. J. Vision **22** (2022). 10.1167/jov.22.8.1 PMC9284468 35816048

[60] E. R. Perl, Ideas about pain, a historical view. Nat. Rev. Neurosci. **8**, 71–80 (2007). 17180164 10.1038/nrn2042

[61] S. Dowiasch, P. Wolf, F. Bremmer, Quantitative comparison of a mobile and a stationary video-based eye-tracker. Behav. Res. Methods **52**, 667–680 (2019). 10.3758/s13428-019-01267-5 PMC7148267 31240632

[62] D. Basu, N. Sendhilnathan, A. Murthy, Neck muscle activity reflects neural patterns of sequential saccade planning in head-restrained primates. J. Neurophysiol. **128**, 927–933 (2022). 36070247 10.1152/jn.00267.2022

[63] V. Navalpakkam, L. Itti, Modeling the influence of task on attention. Vision Res. **45**, 205–231 (2005). 15581921 10.1016/j.visres.2004.07.042

[64] M. F. Land, D. N. Lee, Where we look when we steer. Nature **369**, 742–744 (1994). 8008066 10.1038/369742a0

[65] C. S. Burlingham, D. J. Heeger, Heading perception depends on time-varying evolution of optic flow. Proc. Natl. Acad. Sci. U.S.A. **117**, 33161–33169 (2020). 33328275 10.1073/pnas.2022984117 PMC7776640

[66] N. Mostofi , Spatiotemporal content of saccade transients. Curr. Biol. **30**, 3999–4008.e2 (2020). 32916116 10.1016/j.cub.2020.07.085 PMC7578117

[67] D. E. Tamir, O. V. Komogortsev, C. J. Mueller, “An effort and time based measure of usability” in *WoSQ 2008* (2008).

[68] M. Kümmerer, T. S. A. Wallis, M. Bethge, Information-theoretic model comparison unifies saliency metrics. Proc. Natl. Acad. Sci. U.S.A. **112**, 16054–16059 (2015). 26655340 10.1073/pnas.1510393112 PMC4702965

[69] Niantic Inc. and Nintendo/Creatures Inc./GAME FREAK Inc., Pokémon GO (Mobile game) (2016).

[70] A. B. Tobii, Eye tracker data quality report: Accuracy, precision and detected gaze under optimal conditions–controlled environment (Tobii Pro Glasses 2 firmware v1.61.) (2017).

[71] A. B. Tobii, *Tobii Pro Glasses 2 User’s Manual* (Tobii AB, 2016).

[72] C. S. Burlingham, N. Sendhilnathan, O. Komogortsev, T. S. Murdison, M.J. Proulx, Data and code for the paper “Motor ‘laziness’ constrains fixation selection in real-world tasks”. Open Science Foundation. https://osf.io/f7gc8/. Deposited 6 February 2023. 10.1073/pnas.2302239121 PMC10962974 38470927

