# Supplementary material for: Motor “laziness” constrains fixation selection in real-world tasks
Source: Proc Natl Acad Sci U S A. 2024 Mar 12;121(12):e2302239121. doi: 10.1073/pnas.2302239121 (PMC10962974; doi:10.1073/pnas.2302239121)
Supplement: Supplementary file 1 — Appendix 01 (PDF) [file pnas.2302239121.sapp.pdf]

PNAS

Supporting Information for

Motor "laziness" constrains fixation selection in real-world tasks

Charlie S. Burlingham, Naveen Sendhilnathan, Oleg Komogortsev, T. Scott Murdison, and Michael J. Proulx

Charlie S. Burlingham.
E-mail: cs.burlingham@gmail.com

This PDF file includes:

Supporting text
Figs. S1 to S16
SI References

Supporting Information Text

Saccade direction and amplitude depended strongly on task. Saccade directions were strongly task-dependent (Fig. S16A). Some tasks showed a strong horizontal plane bias as has been observed in some previous studies of saccade statistics during free-viewing (1, 2). Others showed an almost even mix of each cardinal direction, such that vertical saccades were as common as horizontal. This was likely driven by reading behavior in the Browsing and Restaurant task, when observers read text on a tablet or phone and scrolled. In other cases (Grocery, Lego), the distribution was more rounded, with more oblique angle saccades than in the other tasks.

Average saccade amplitudes ranged from 5-10º between tasks (Fig. S16B). This is consistent with past findings that saccade amplitudes tend to be under 10º on average. Saccades were smallest in the browsing task and largest in the grocery task. In the browsing task, participants mostly read text on their phone from a distance, generating small saccades. In the grocery task, participants often searched drawers while standing, such that their heads were angled down, then looked up and searched the room for other items, generating large saccades coupled with head rotation. We performed two-sided permutation tests on every pair of tasks, and 34/36 comparisons were significant following false discovery rate (FDR) correction. That is, nearly all differences in amplitude observed between tasks were statistically significant.

No evidence of a systematic negative relationship between head pitch and gaze-in-head position. In certain tasks, gaze-in-head fixation position and duration distributions were vertically offset from the head orientation by a few degrees (Fig. S1, S2). This raises the possibility that some participants pointed their heads up or down and viewed the scene with their eyes in an offset position, vs. looking up or down with their head level with gravity. This would cause a systematic negative correlation between the gaze-in-head position and head pitch. However, we did not find evidence for this relationship. We ran a linear mixed effects model predicting the vertical gaze-in-head position based on head pitch, including random intercepts and slopes for each recording session and task to account for differences between sessions in head pitch. The relation between head pitch and gaze-in-head position was non-significant ($p = 0.62$, $N = 4{,}792{,}143$ gaze/head orientation samples), and the power was high given the large N. Of course, during specific cases like VOR, we should expect a nearly perfect negative correlation between the head orientation and gaze-in-head position, but this shouldn't be expected to dominate the preferred gaze-in-head position over an entire experiment (also, our fixation detector purposely attempted to exclude periods of VOR).

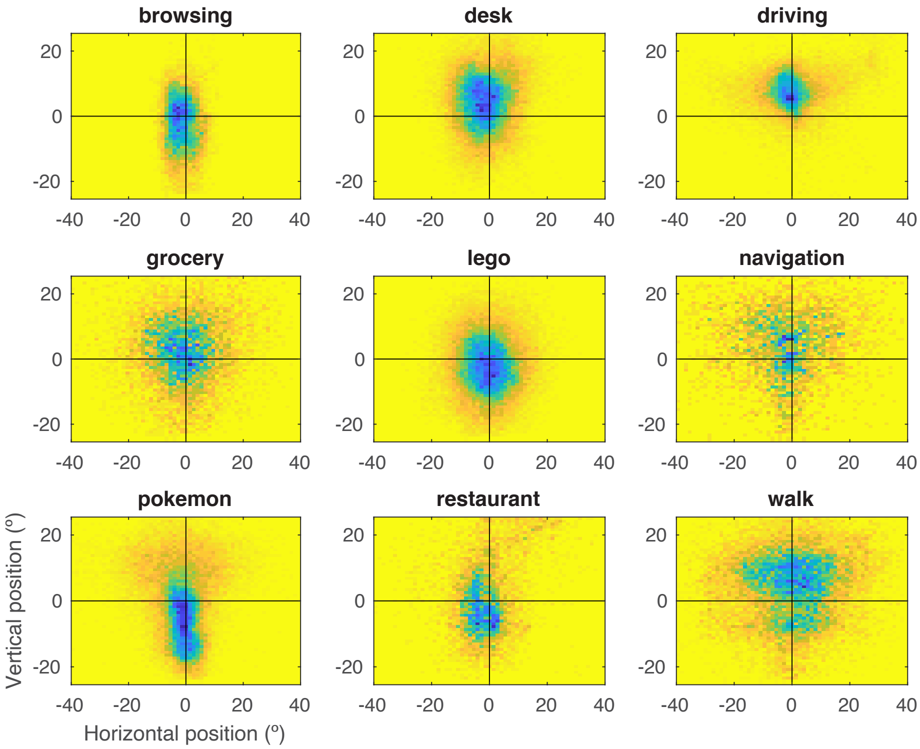

Fig. S1. Probability distributions of fixation location for each task. Same format as Fig. 1, bottom panel. Colorbar limits were different for each subpanel, given that the amount of data varied between tasks.

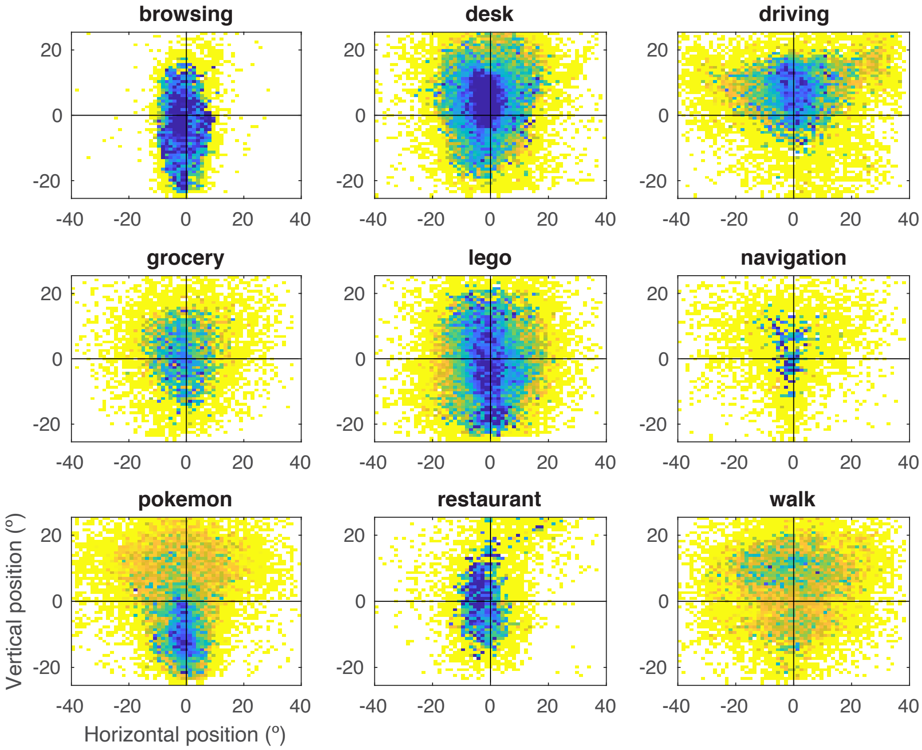

Fig. S2. Spatial map of average fixation duration for each task. Same format as Fig. 1, middle panel. Colorbar limit is the same for each subpanel and was chosen to be consistent with Fig. 1. Note that some data points are above 0.8 s, for example in the browsing and desk tasks, but will be plotted at the darkest shade of blue available. White pixels are spatial bins for which there was only one data point / fixation or fewer.

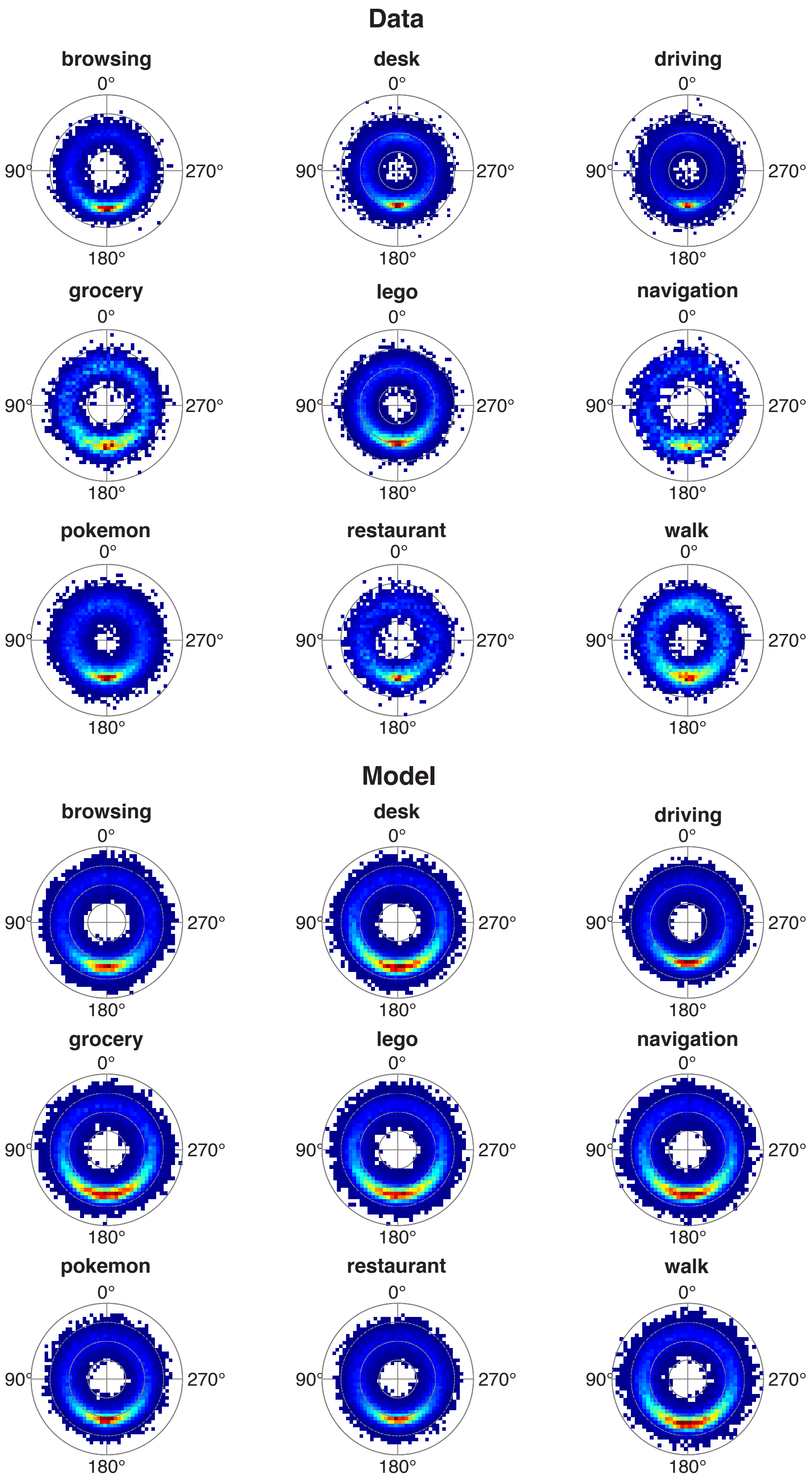

Fig. S3. Relative saccade direction and amplitude joint distributions for each task, data versus model. Same format as Fig. 2 but for each task.

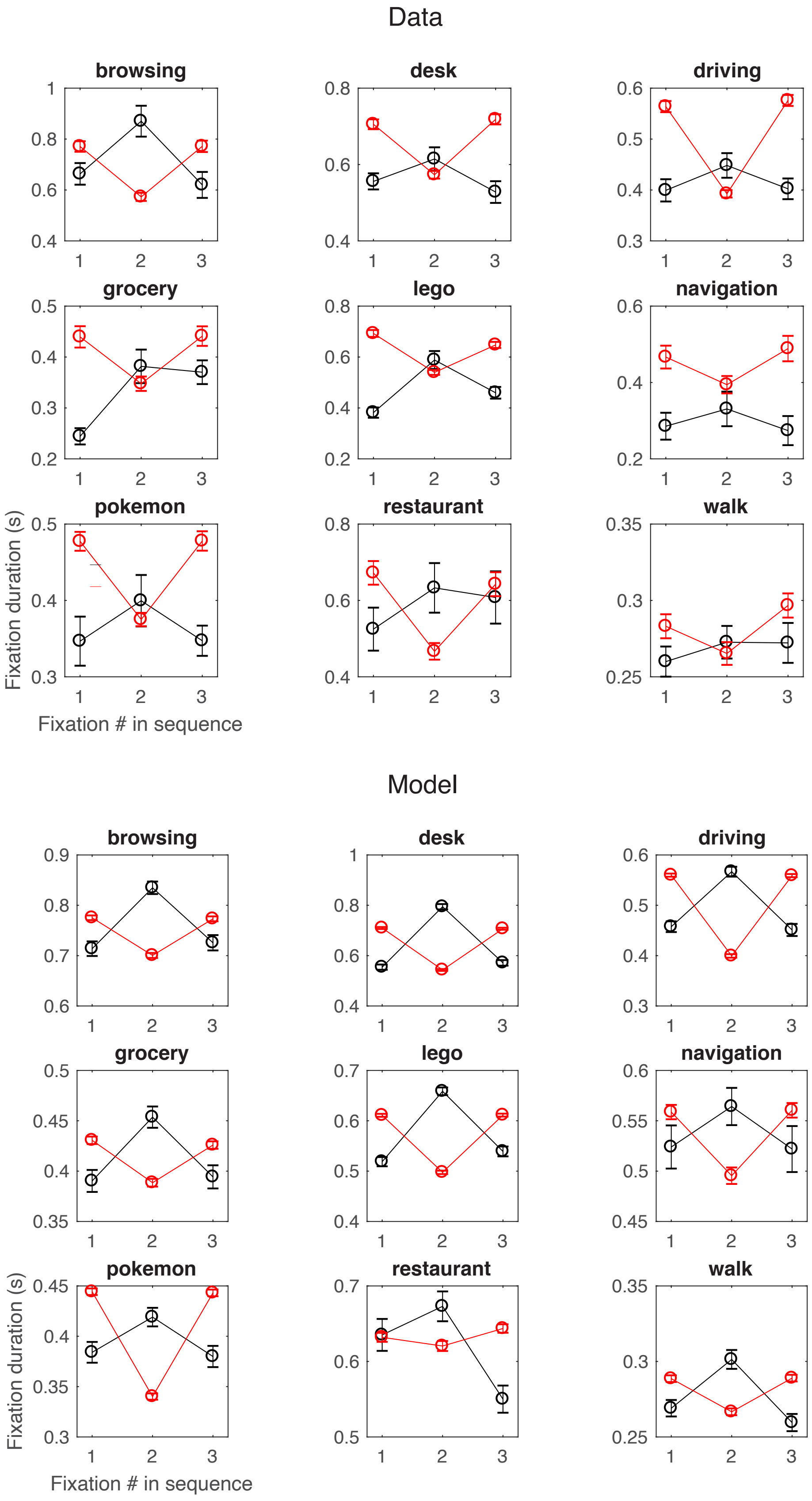

Fig. S4. Fixation duration for three-fixation sequences for each task, data versus model. Same format as Fig. 3 but for each task.

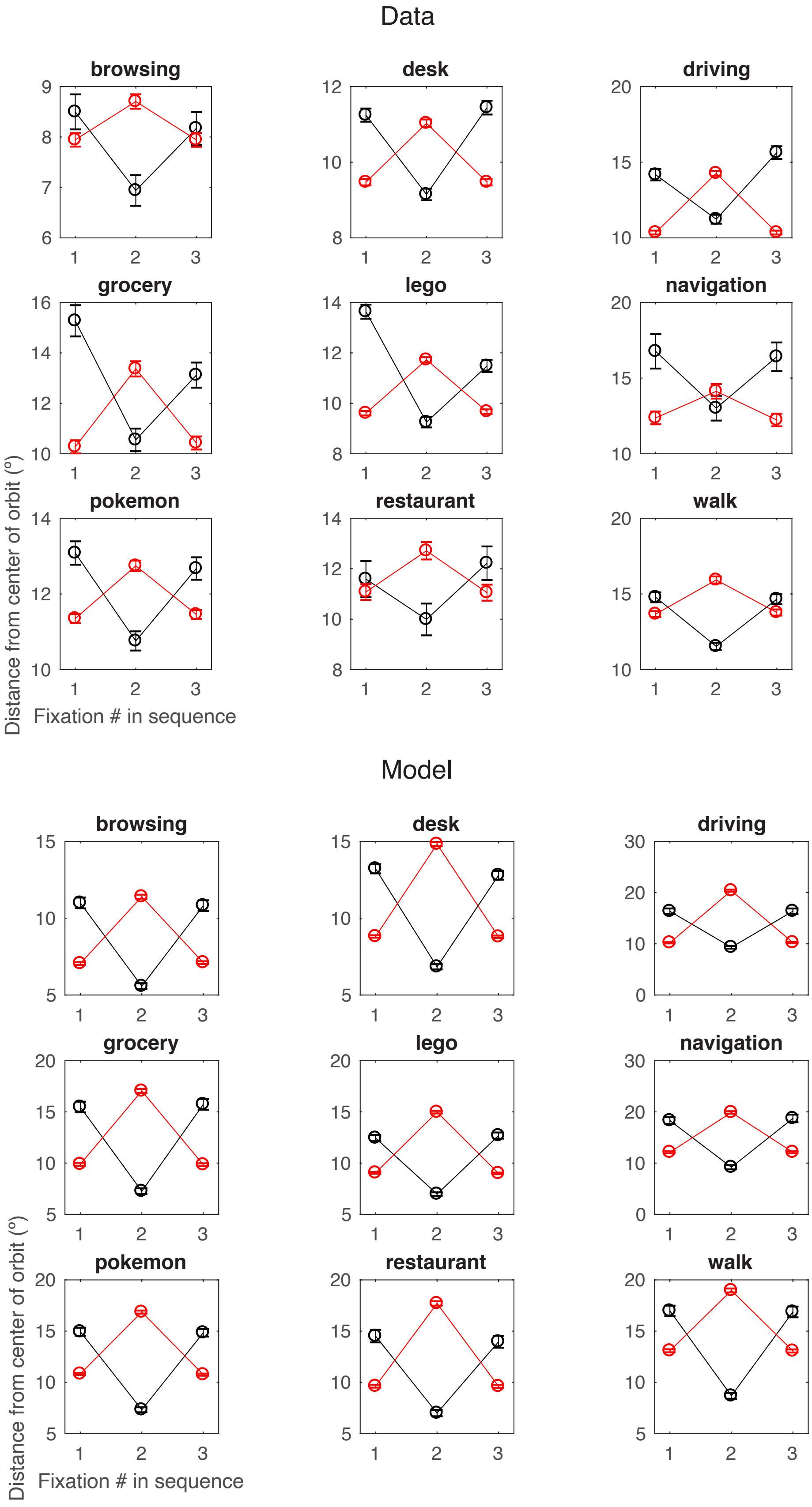

Fig. S5. Distance from center of FOV for three-fixation sequences for each task, data versus model. Same format as Fig. 3 but for each task.

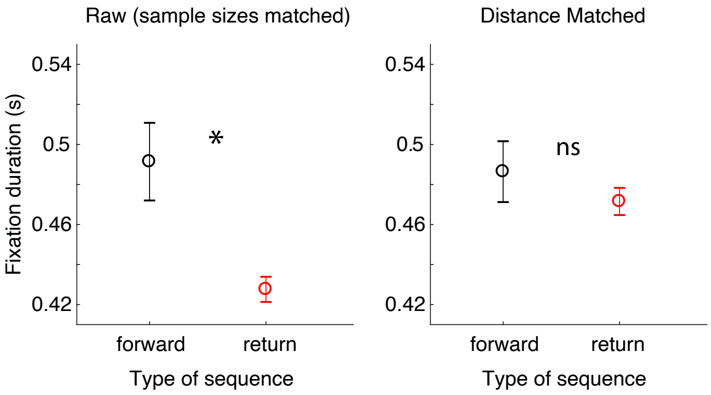

Fig. S6. Control analysis: duration effects disappeared when distance from the center of FOV was fixed. Panel 1, average fixation duration in seconds preceding a forward or return saccade. Same data as shown in Fig. 3 but resampled such that sample sizes are equivalent in panels 1 and 2 (i.e., equivalent N after distance thresholding has been applied). The mean p-value across 100 boostrap resamples was 0.0035 and 98% of p-values were < 0.05 (N permutations for each test = 1000; one-tailed, forward > return). Panel 2, duration of fixation preceding forward or return saccade, after matching distance from the center of the FOV for forward and return sequences, not significant (mean p-value = 0.2383, N bootstraps = 100; N permutations per bootstrap = 1000; one-tailed, forward > return; only 24 % of p-values were < 0.05). Distance-matching consisted of finding sequences that were within 2º of the median of the distribution of distances (i.e., where there was the most data). Distance matching was done for each participant and task separately. All plots depict pooled data across all recordings.

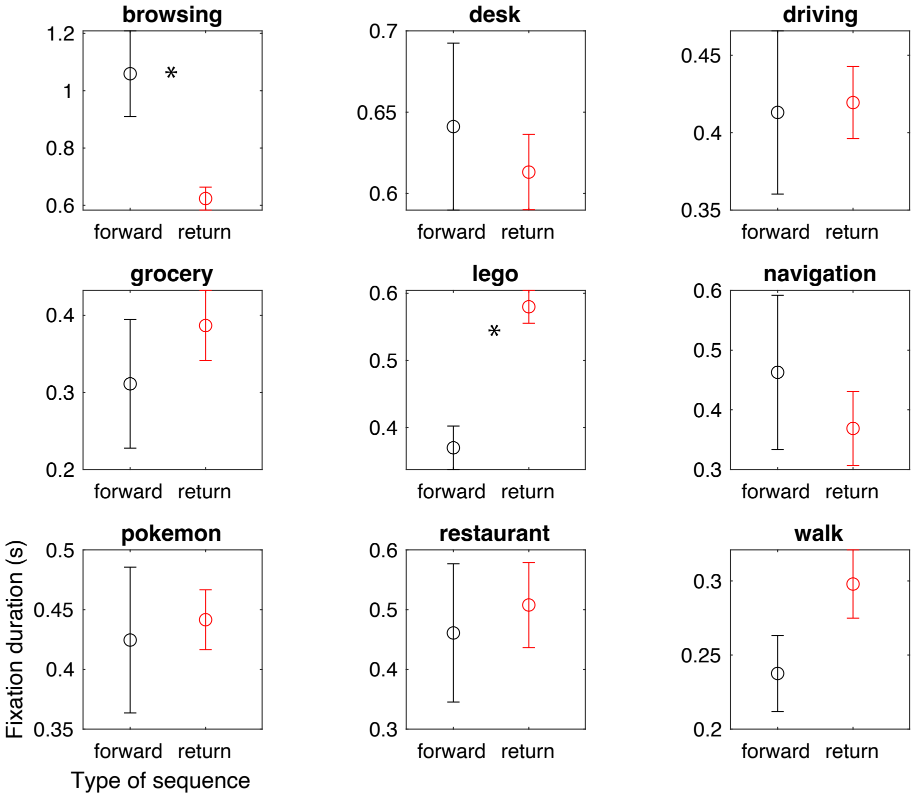

Fig. S7. Control analysis for each task. Same format as Fig. S6, right subpanel, where distances were matched, except for each task separately. Asterisks represent outcome of two-tailed permutation tests, 1000 permutations each. P was > 0.05 for each task except Browsing ($p = 0.002$) and Lego ($p = 0.001$).

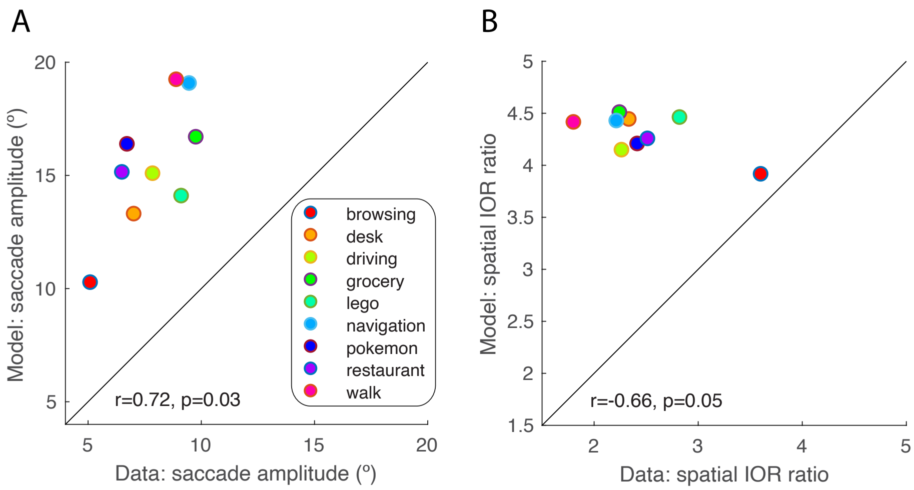

Fig. S8. A random sampling model overestimates both saccade amplitude and the ratio of occurrence of return vs. forward saccades by around a factor of two. A. Median saccade amplitude (º) for the data versus the model, in each task. A model based on random sampling of the fixation density captures task dependency in saccade amplitude, hence the significant correlation, but overestimates amplitudes by around a factor of two. This overestimation is consistent with previous reports (Bays & Hussain, 2012), and demonstrates that people make smaller saccades than would be expected by the time-integrated statistics of their gaze locations. B. Spatial IOR ratio, the ratio of return to forward saccade occurrence, defined by slicing the relative saccade amplitude/direction joint distribution into four equal quadrants defined by the oblique angles and comparing the density in the two quadrants centered on precise return and forward saccades. The model overestimates this ratio by around a factor of two and doesn't capture task dependency in the ratio.

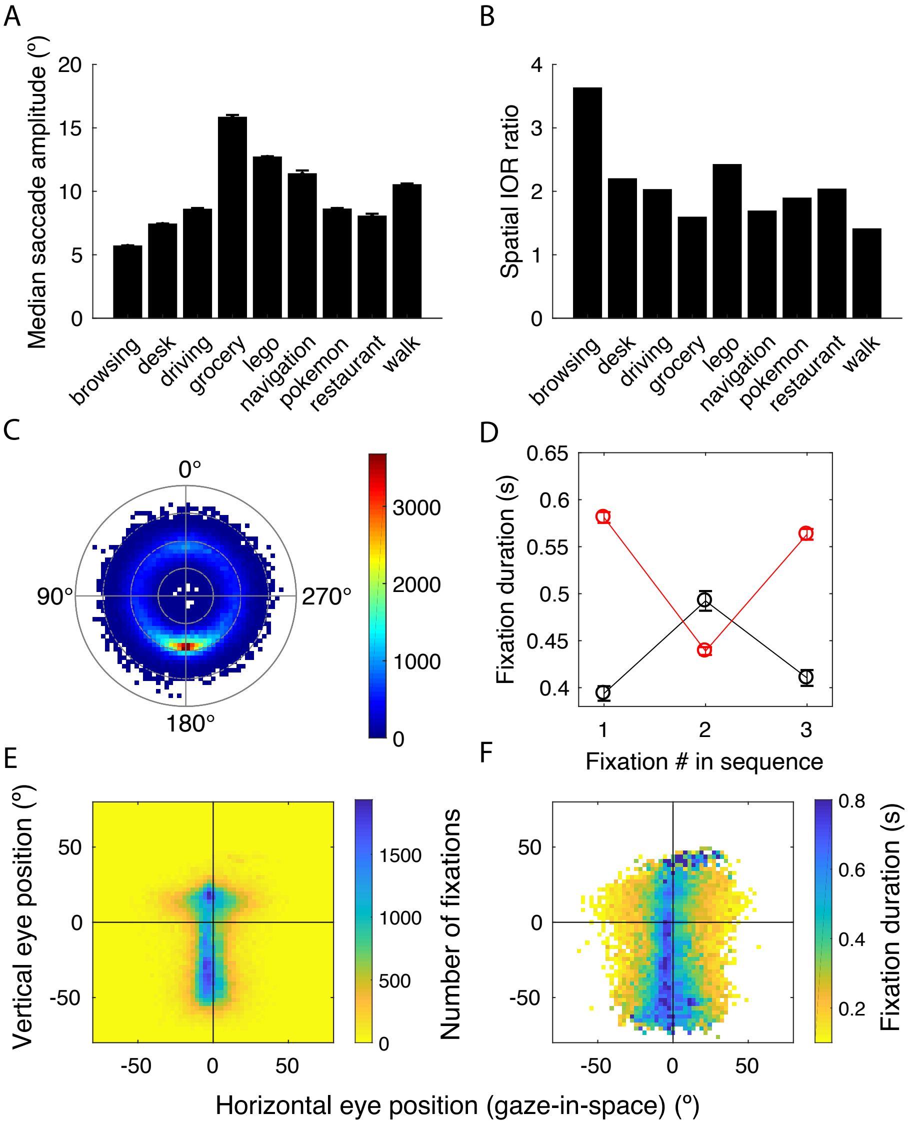

Fig. S9. Possible artifactual influences of head movement on gaze position do not explain our findings: gaze-in-body summary / pooled analyses. A-F, same format as prior figures but in gaze-in-body coordinates, (i.e., after counter-rotating gaze-in-head positions according to momentary head rotation estimates derived from the onboard inertial measurement unit [IMU]). See prior figure captions for detailed explanations. A. Saccade amplitude (º). B. Spatial IOR ratio. Relative saccade amplitude and directions (overall Spatial IOR ratio before vs. after accounting for head movements: 2.43 vs. 2.09). D. Temporal sequences of fixation duration for forward and return saccades (same format as Fig 3). For no individual task was the second fixation in a return sequence significantly longer than the second fixation in a forward sequence (one-tailed permutation tests, $p > 0.05$ for each task, 1000 iterations), i.e., we didn't find find evidence consistent with temporal IOR in any task. E. Probability distribution of fixation position. F. Spatial map of average fixation duration (note that only bins with greater than 2 data points are shown in color, to reduce sampling error while also showing rough shape of function). Note that the axis limits are much larger than in Fig. 1, reflecting the impact of head movements during task on fixation position in body-centered coordinates.

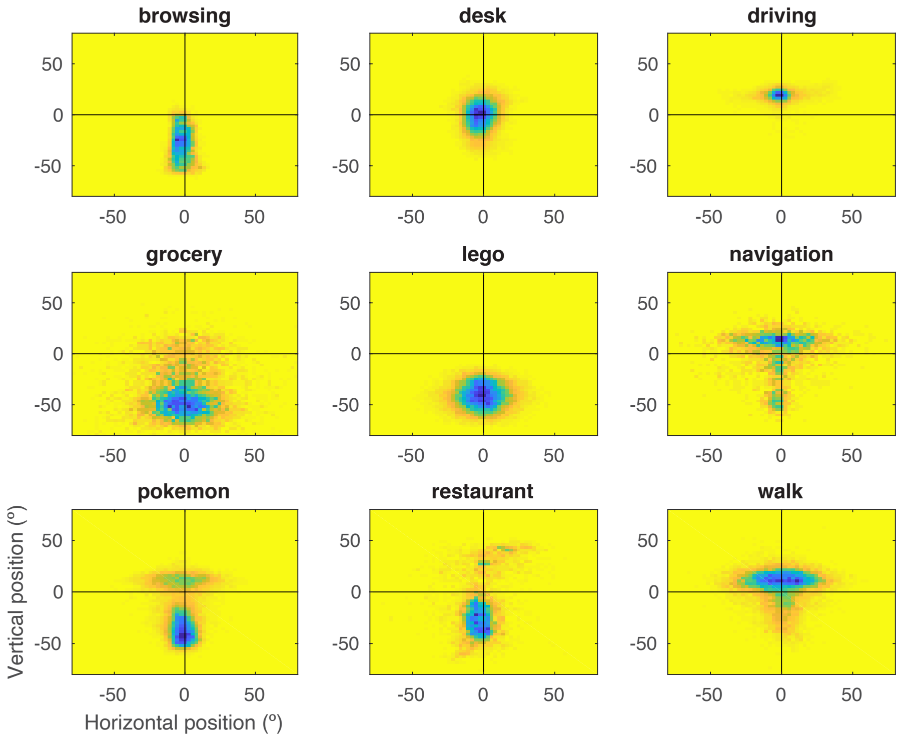

Fig. S10. Fixation position (gaze-in-body) for each task. Same format as Fig. S1 but for gaze-in-body data. Note the axis limits are much larger. These body-centered distributions were 1.65x more dispersed on average across tasks than the head-centered ones (dispersion measured as the sum of the vertical and horizontal standard deviations). The body:head-centered ratios of dispersion for each task were: 1.65, 1.4, 1.38, 2.13, 1.45, 1.69, 1.81, 1.96, 1.42; order of tasks: Browsing, Desk, Driving, Grocery, Lego, Navigation, Restaurant, Pokémon, Walk.

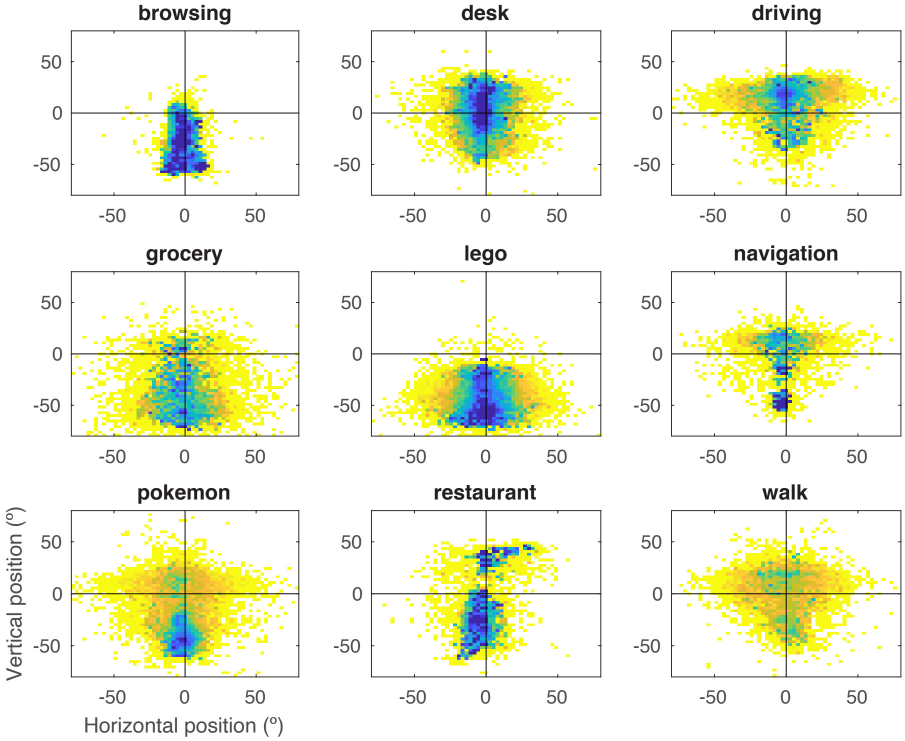

Fig. S11. Fixation duration (gaze-in-body) for each task. Same format as Fig. S2 but for gaze-in-body data. Note the axis limits are much larger.

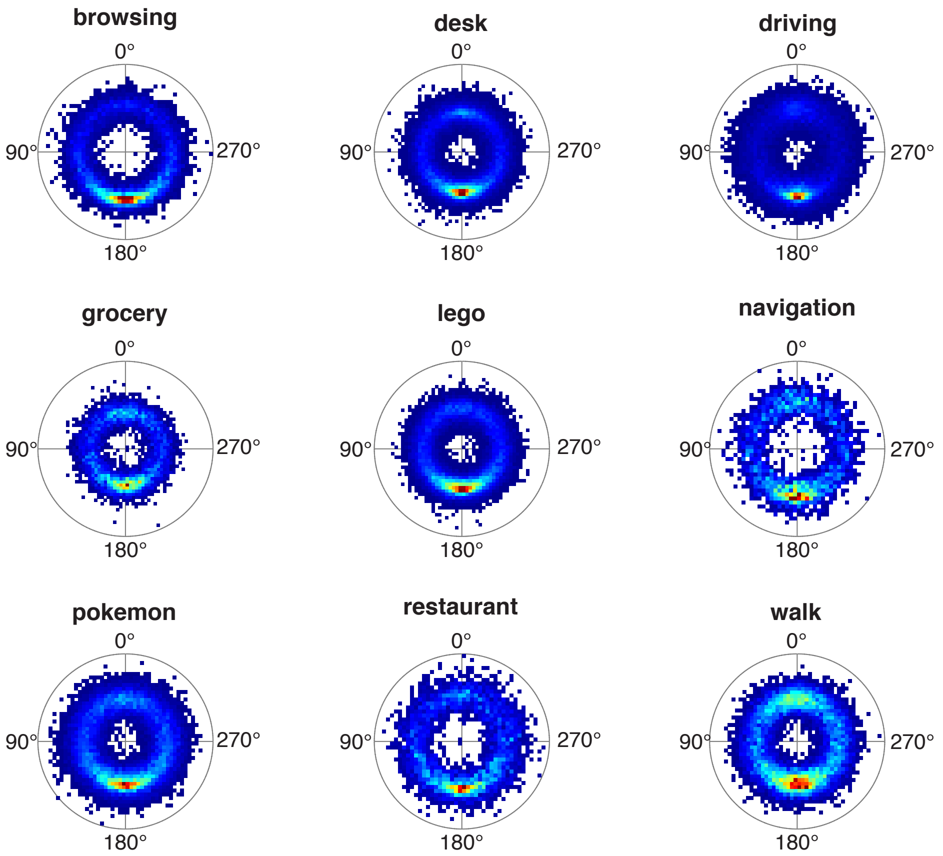

Fig. S12. Relative saccade angle and amplitude joint distributions (gaze-in-body) for each task. Same format as Fig. S3 but for gaze-in-body data.

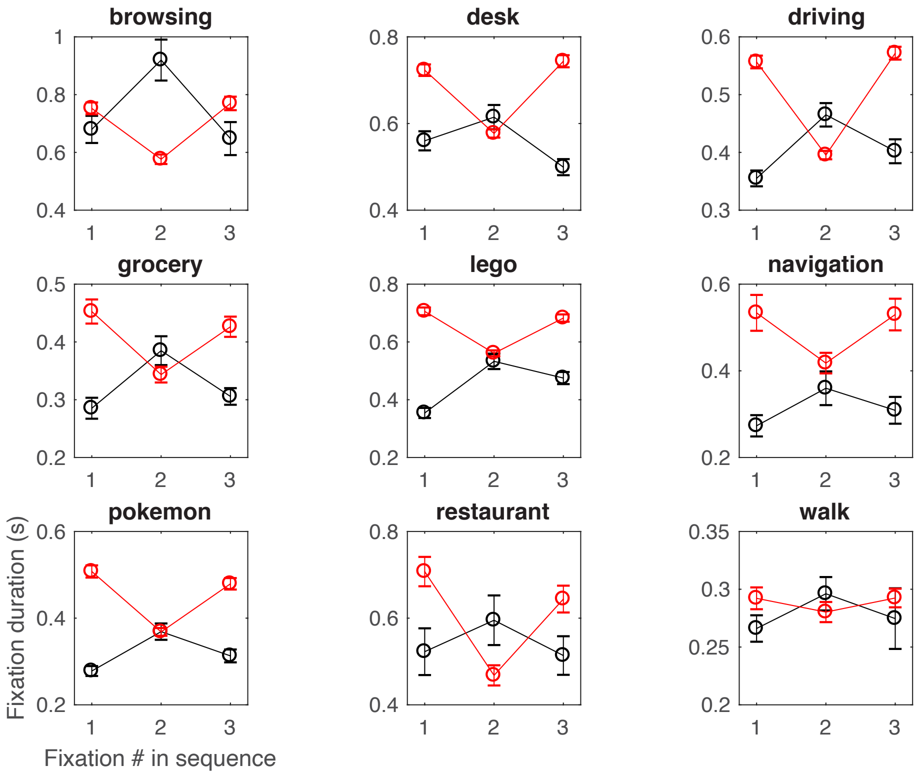

Fig. S13. Fixation duration sequences (gaze-in-body) for each task. Same format as Fig. S4 but for gaze-in-body data. Note the axis limits are different (in some subpanels). In no task did we find that the fixation #2 in the sequence was significantly longer for return than forward sequences (i.e., consistent with temporal IOR). There was a visible IOR-like trend for the Navigation task, however, it must be interpreted in the context of the surrounding inverted 3-fixation sequences, which were prominent.

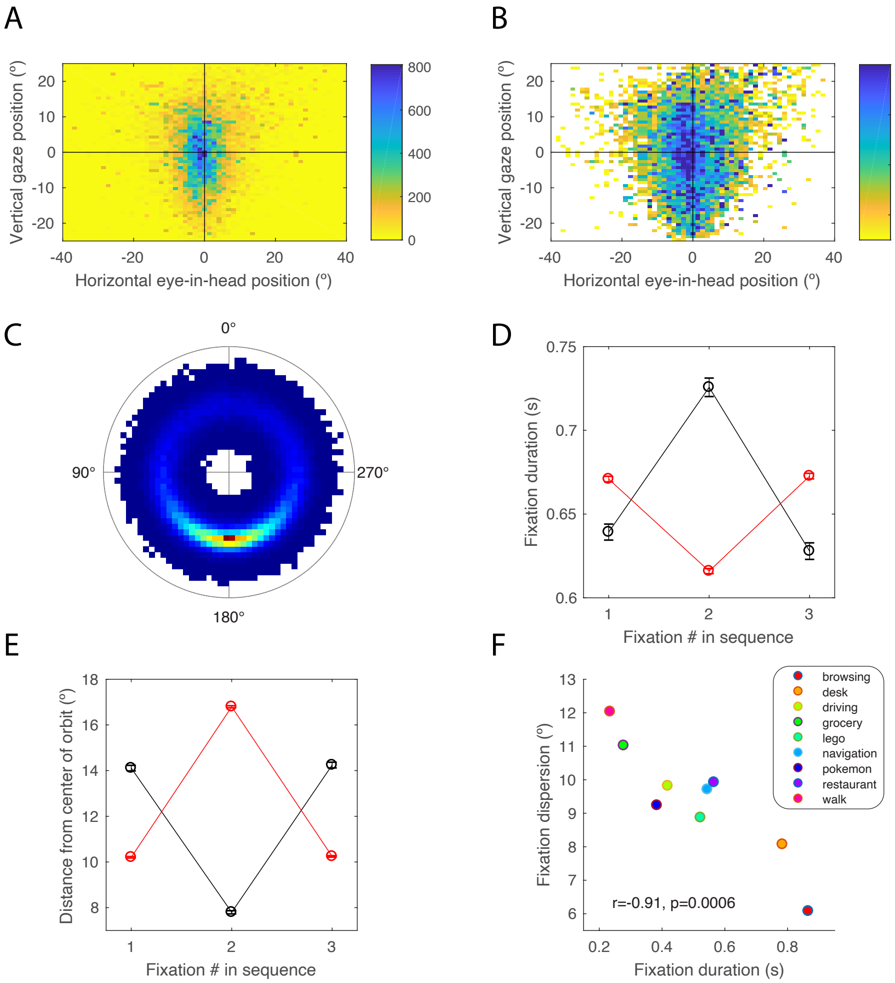

Fig. S14. Possible artifactual influences of head movement on gaze position do not explain our findings: gaze-in-head position/duration statistics are similar during periods of low head movement. Panels A and B, same format as Fig. 1, but only for fixations when head movement angular speed was less than 3 º/s. Panels C-F, model predictions based on simulated fixations from per-task distributions for periods when head movement was low (i.e., same format as Fig. 2-4, model predictions). This shows that the model predicts similar results as seen in original data, for periods of low head movement. Note that in panels A and B, the Walk task was removed, because N after thresholding for head speed was low, causing the maps to be dominated by sampling error.

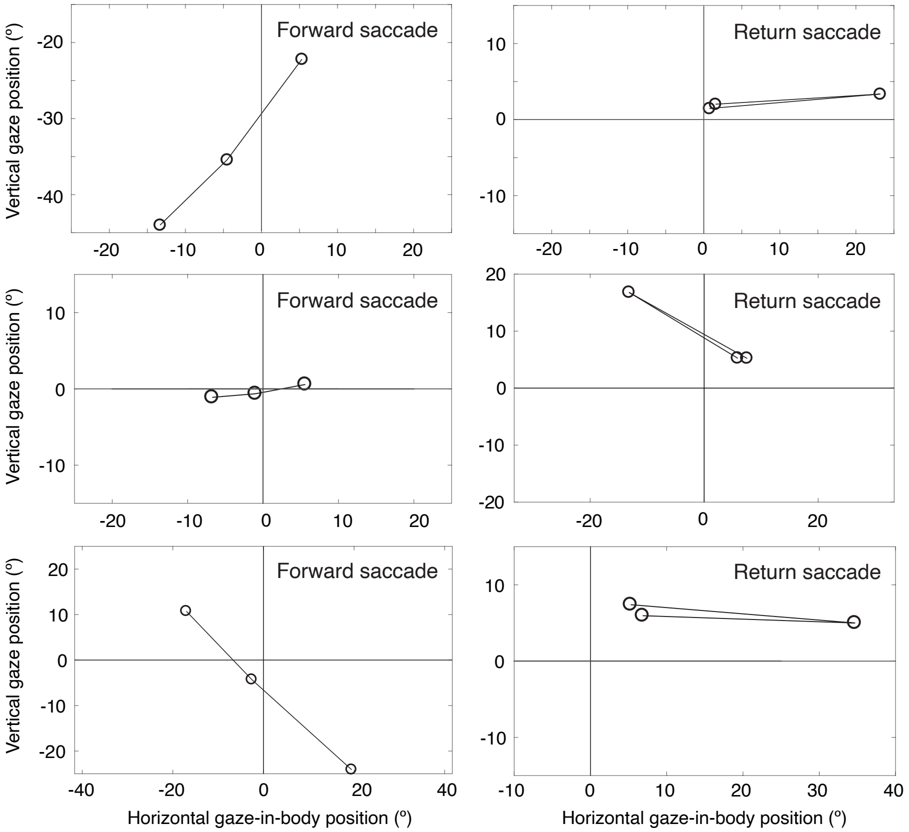

Fig. S15. Examples of forward and return saccades from the data. Note the different axis limits for each plot.

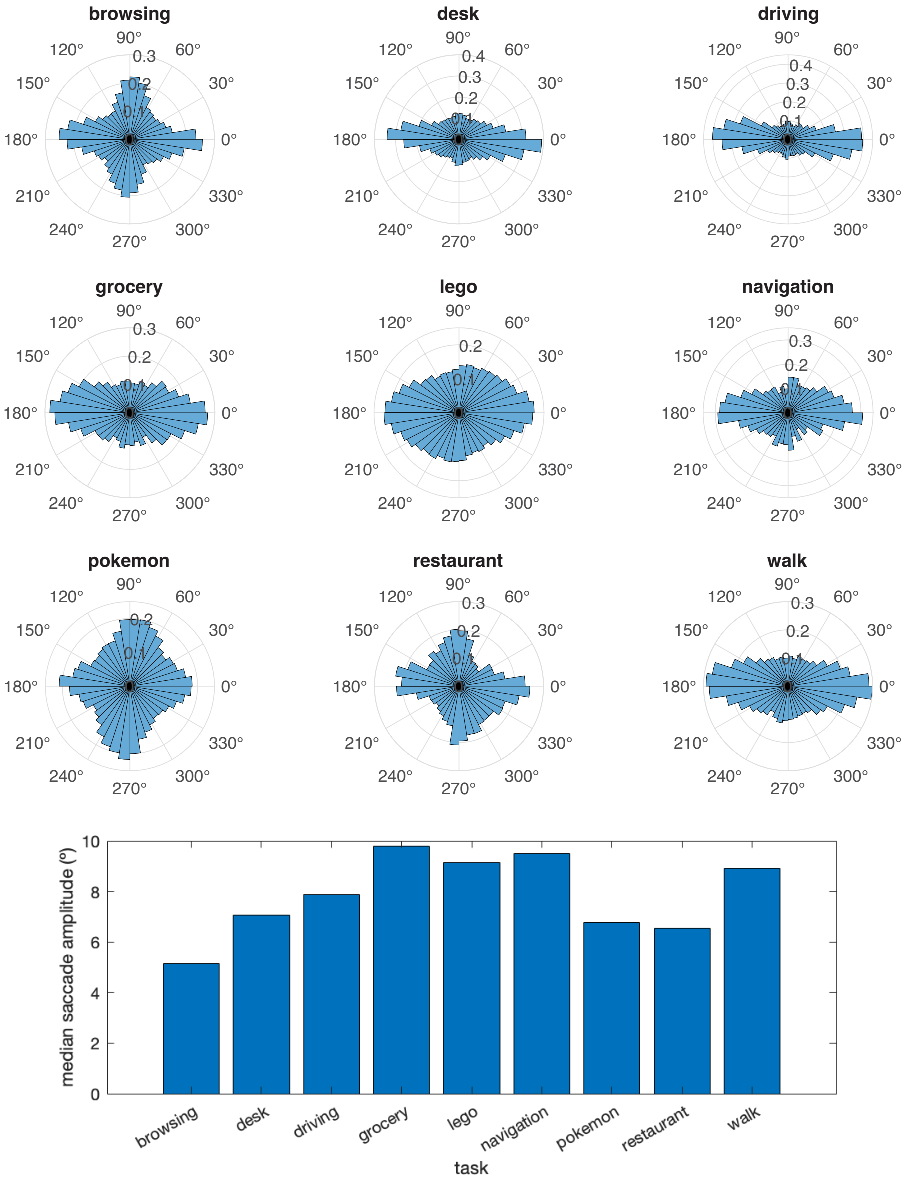

Fig. S16. Saccade direction distributions and median amplitudes for each task (head-centered coordinates). A. Saccade direction histograms for each task. Radius indicates probability and polar angle indicates saccade direction (where 90º is vertical). B. Median saccade amplitude (º) for each task. Median is plotted instead of the full distributions because the distribution shape is similar for each task and quite broad so it's difficult to tell where the center is by eye, even in log space.

References

1. PM Bays, M Husain, Active inhibition and memory promote exploration and search of natural scenes. *J. vision* **12 8** (2012).
2. OL Meur, Z Liu, Saccadic model of eye movements for free-viewing condition. *Vis. Res.* **116**, 152–164 (2015).
